# Attenuated clinical and osteoclastic phenotypes of Paget’s disease of bone linked to the p.Pro392Leu/*SQSTM1* mutation by a rare variant in the *DOCK6* gene

**DOI:** 10.1186/s12920-022-01198-9

**Published:** 2022-03-03

**Authors:** Mariam Dessay, Emile Couture, Halim Maaroufi, Frédéric Fournier, Edith Gagnon, Arnaud Droit, Jacques P. Brown, Laëtitia Michou

**Affiliations:** 1 CHU de Québec-Université Laval Research Centre, Quebec City, QC Canada; 2 Institut de Biologie Intégrative Et Des Systèmes (IBIS), Université Laval, Quebec, QC Canada; 3 Department of Medicine, Université Laval, Quebec, QC Canada; 4 Department of Rheumatology-R4774, CHU de Québec-Université Laval, 2705 boulevard Laurier, Quebec, QC G1V 4G2 Canada

**Keywords:** Paget’s disease of bone, *SQSTM1* gene, *DOCK6* gene, p.Pro392Leu mutation, p.Val45Ile variant, Whole exome sequencing, Serum response factor

## Abstract

**Background:**

We identified two families with Paget's disease of bone (PDB) linked to the p.Pro392Leu mutation within the *SQSTM1* gene displaying a possible digenism. This study aimed at identifying this second genetic variant cosegregating with the p.Pro392Leu mutation and at characterizing its impact on the clinical and cellular phenotypes of PDB.

**Methods:**

Whole exome sequencing was performed in one patient per family and two healthy controls. We compared clinical characteristics of PDB in 14 relatives from the two families. The osteoclastic phenotype was compared in in vitro differentiated osteoclasts from 31 participants carrying the *DOCK6* and/or *SQSTM1* variants. Tridimensional models of SQSTM1 and DOCK6 proteins were generated to evaluate the impact of these variants on their stability and flexibility. Statistical analyses were performed with Graphpad prism.

**Results:**

Whole-exome sequencing allowed us to identify the p.Val45Ile missense variant in the *DOCK6* gene in patients. In both families, the mean age at PDB diagnosis was delayed in pagetic patients carrier of the p.Val45Ile variant alone compared to those carrying the p.Pro392Leu mutation alone (67 vs. 44 years, *P* = 0.03). Although both p.Val45Ile and p.Pro392Leu variants gave rise to a pagetic phenotype of osteoclast versus healthy controls, the p.Val45Ile variant was found to attenuate the severity of the osteoclastic phenotype of PDB caused by the p.Pro392Leu mutation when both variants were present. The *DOCK6* mRNA expression was higher in carriers of the p.Val45Ile variant than in pagetic patients without any mutations and healthy controls. Structural bioinformatics analyses suggested that the p.Pro392Leu mutation might rigidify the UBA domain and thus decrease its possible intramolecular interaction with a novel domain, the serum response factor–transcription factor (SRF-TF)-like domain, whereas the p.Val45Ile variant may decrease SRF-TF-like activity.

**Conclusion:**

The p.Val45Ile variant may attenuate the severity of the clinical phenotype of PDB in patient carriers of both variants. In vitro, the rare variant of the *DOCK6* may have a modifier effect on the p.Pro392Leu mutation, possibly via its effect on the SRF-TF-like.

**Supplementary Information:**

The online version contains supplementary material available at 10.1186/s12920-022-01198-9.

## Background

Paget's disease of bone (PDB) is a late-onset focal chronic bone disorder which may be asymptomatic [1]. PDB is slightly more prevalent in men than in women and the prevalence of PDB increases with age [2]. This disorder is characterized by the gradual replacement of normal bone tissue by chaotic and poor quality tissue with anarchic structure [1, 3]. The pathophysiology of PDB remains poorly understood, but both genetic and environmental factors are involved in its pathogenesis [4, 5]. The p.Pro392Leu mutation in the *SQSTM1* gene is the most frequently reported mutation, up to 46% of familial forms of PDB in the French-Canadian population, with high penetrance, greater than 80% after 60 years [6–8]. After the initial description of the p.Pro392Leu mutation in PDB, 27 additional mutations were reported in the UK, Australia, New Zealand, the USA, the Netherlands, Italy, France and China in patients with PDB, giving rise to more than 20 different amino acid substitutions and various ubiquitin-associated domain (UBA) truncations [9]. The *SQSTM1* gene encodes for the protein P62, which is involved in the NF-κB signaling pathway, apoptosis, activation of Nrf2 and macroautophagy [10, 11]. The p.Pro392Leu mutation in the *SQSTM1* gene was reported to increase both osteoclastogenesis and the osteoclastogenic potential of bone microenvironment [12, 13]. Genome-wide association studies allowed the identification of other susceptibility loci of PDB in genes such as *RIN3, CSF1*, *OPTN, TM7SF4, TNFRSF11A* as well as *PML, CASR, ESR1, TNFRSF11B, VCP,* and *CTHRC1* [14–17]. Genetic variants of the *CSF1, OPTN, DCSTAMP* and *TNFRSF11A* genes were reported to represent 67% of the genetic risk of PDB [18]. Several environmental factors associated with PDB might have contributed to the decrease in prevalence and reduced clinical severity observed in some countries [19]. For instance, poor calcium and vitamin D intake, and consumption of uncontrolled beef meat has been associated with PDB [20]. Other environmental factors such as heavy metals like arsenic or lead may also have contributed to PDB pathogenesis [5, 21, 22]. The observation of intracellular inclusions in osteoclasts possibly linked to measles virus nucleocapsids (MVNP) gave rise to the hypothesis of a possible persistent viral infection [1, 23]. MVNP and the p.Pro392Leu mutation within the *SQSTM1* gene may both contribute to promote osteoclastogenesis according to clinical studies and in vivo studies in transgenic mices [24], suggesting that genetic and/or environmental modifier factors may regulate the expression of the *SQSTM1* gene.

**Fig. 1:**
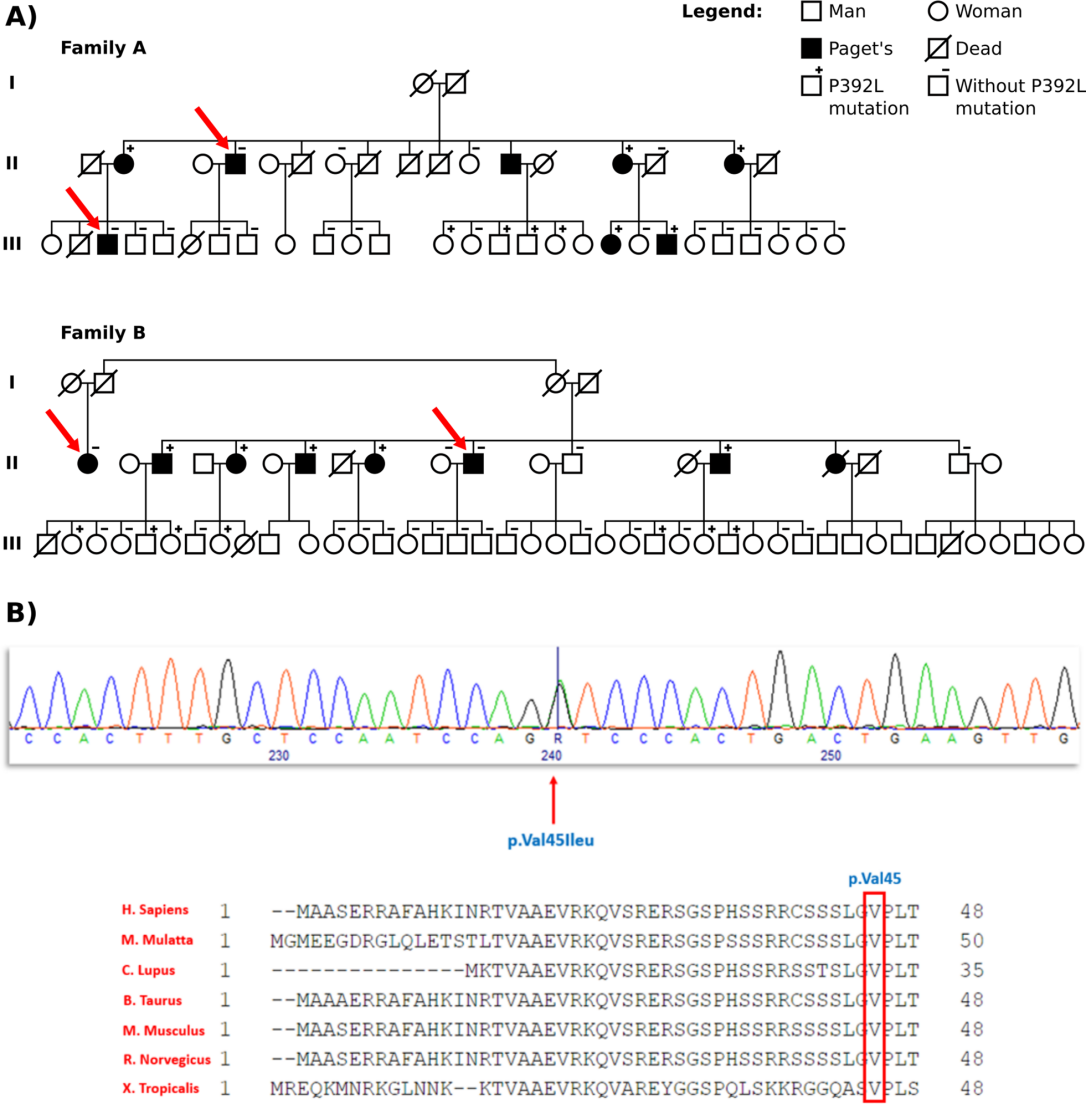
**A** Pedigrees of the two families studied by whole exome sequencing. The red arrow targets the participants to the whole exome analysis. **B** The p.Val45Ile mutation sequence and conservation during evolution of the amino acid valine in position 45 in *DOCK6* gene, according to Homologene

In our cohort, we have identified two large families in which patients with PDB linked to the p.Pro392Leu mutation coexist or not in the same sibship (Fig. 1A). We therefore hypothesized a possible digenism, namely that a variant in a second gene, less penetrant than the p.Pro392Leu mutation, could explain the presence of these phenocopies in both families. This study aimed at identifying this possible second genetic factor that may cosegregate in the two familial forms of PDB linked to the *SQSTM1* gene mutation described above, and at determining the impact on the clinical and cellular phenotypes of this rare variant and its modifier effect on the p.Pro392Leu mutation.

## Methods

### Recruitment of participants

This study was approved by the CHU de Québec-Université Laval Ethics Committee (IRB numbers MP-20-2014-1820, B13-11-1820) and all participants signed a consent form before inclusion in the study. We studied 62 participants, including patients with PDB, from familial and non-familial forms, as well as healthy controls from the French-Canadian cohort. Among the 62 participants, 25 participants were part of two large families, family A and B whose pedigrees are shown in the Fig. 1. From these two families, six participants were studied for exome sequencing, 14 for the clinical phenotype and 13 for the cellular phenotype. All participants lived in the same geographic area surrounding 120 km of Quebec City.

### Whole exome sequencing

To identify a possible second variant cosegregating with the p.Pro392Leu mutation in the two large pedigrees of PDB described above, we performed whole exome sequencing in two relatives with PDB but not mutated in the *SQSTM1* gene, who appeared as phenocopies in each family, as well as in two healthy controls. Genomic DNA from these six samples was prepared using the Agilent SureSelect XT Human All exons V5 (Agilent Technologies, Santa Clara, USA). 3.0 μg of genomic DNA was fragmented on a Covaris instrument (Covaris, Woburn, MA, USA). Fragments of 150–200 bp were purified and checked for quality control using a Tapestation 2200 instrument (Agilent Technologies, Santa Clara, USA). Library preparation was completed using standard sample preparation protocol: end repair, adenylation, ligation of paired-end adaptors. After adaptor ligation and purification using Agencourt AMPure XP beads (Beckman Coutler, Missisauga, Ontario, Canada), libraries were amplified for six cycles in the pre-capture amplification step. Following hybridization to the target-specific capture library and purification using streptavidin-coated beads (Dynabeads MyOne Streptavidin T1, ThermoFisher Scientific, Canada), libraries were amplified for 12 cycles with indexing primers. The quality of final amplified libraries was examined on a Tapestation 2200 instrument to check library size (250–350 bp) and the quantification was done on QuBit 3.0 fluorometer (ThermoFisher Scientific, Canada). Subsequently, exome libraries with unique index were pooled together in equimolar ratio and the pool was sequenced using one lane of a high ouput flowcell on an Illumina HiSeq 2500 V4 system at the Next-Generation Sequencing Platform, Genomics Centre, CHU de Québec-Université Laval Research Centre, Quebec City, Canada for paired-end 125 bp sequencing.

### Variants filtering and bioinformatic analyses

For the bioinformatics analysis, we made a filtering of the variants according to the frequency of the minor allele below 1%. Although we generated in silico prediction of the variant effect (deleterious or not) based on several database, we did not used these data as a filter for the variant selection. Indeed, the p.Pro392Leu mutation of the *SQSTM1* gene, which is the most frequent variant linked to PDB, was in silico predicted as benign or tolerated at the time of its discovery. This filtering was followed by the intra-family segregation analysis within the two families. Due to the founder effect of the French-Canadian population, we expected that phenocopies of both families were more likely to share the same modifier variant of the p.Pro392Leu mutation. To search for possible interactions of the variants retrieved by the whole exome analysis in our two families with the *SQSTM1* gene, an in silico analysis of gene interaction was performed using the GeneMANIA software (https://genemania.org/). Following intra-familial segregation analysis, to select candidate genes for the targeted sequencing, we also reviewed the literature data on predicted gene and protein functions, from database such as Pubmed, Genecards, OMIM.

### Targeted sequencing

Targeted sequencing was performed in 18 patients with PDB from four other families in which we had one patient with PDB not carrier of the p.Pro392Leu mutation and at least one patient with PDB carrier of this mutation. The targeted sequencing was performed on 106 genes using next generation sequencing. Eighteen libraries were prepared using a custom design Agilent SureSelect XT target Enrichment kit (Agilent Technologies, Santa Clara, USA). The design included 106 genes (exons and intron/exon splice junctions) for a size capture of 498.73 Kb. Library preparation was done according to the same protocol as whole exome sequencing with the exception of five libraries for which 200 ng of genomic DNA were studied. The libraries were amplified for 16 cycles instead of 12 with indexing primers. The checking library size was (325–450 bp) and high output flowcell on an Illumina HiSeq 2500 V3 system at the Next-Generation Sequencing was used.

### Clinical phenotype

At baseline, we collected data on the age at PDB diagnosis, the number of affected bones and the level of tALPs (expressed as the number of time the midpoint of normal range) in 14 patients carrying the p.Pro392Leu and/or the p.Val45Ileu variant, all were issued from our two informative families, of which four patients were studied by whole exome sequencing.

### Cell cultures

Cellular phenotype was studied in 27 participants including some relatives from the two families investigated in the whole exome analysis, 18 of whom carried the p.Pro392Leu mutation alone or the novel rare variant alone or both variants, five patients carried neither of the two variants and four healthy controls not carrier of either variant. We isolated peripheral blood mononuclear cells (PBMCs) from 50 ml of peripheral blood of each participant by density gradient centrifugation using the Ficoll-Paque method. The cells were counted using the Bio-Rad TC20 automatic cell counter and seeded on a 12-well plate with 1.1 ml of suspended cells/well (3.3 × 10^6^ cells per well) for the RNA extraction and the protein expressions on the cell lysates. The in vitro differentiation of PBMCs into osteoclasts was carried out using 60 ng/ml of RANKL (Peprotech, Rocky Hill) and 25 ng/ml of hMCSF (eBioscience, San Diego) during 21 days. The cells were cultured under the optimum condition of 37ºC with 5% CO2 using Alpha-MEM medium, which contains 10% FBS + 1% Penicillin–Streptomycin (Wisent, St-Bruno, QC, Canada). Mediums were changed every 3–4 days. Immunofluorescence assay was performed using Lab-Tek (Fisher Scientific, Ottawa) in which we seeded 1.2 × 10^6^ cells. To assess bone resorption abilities in mature osteoclasts, we seeded 10^6^ cells per well on an Osteoassay plate (Fisher Scientific, Ottawa).

### Immunofluorescence assay

After 21 days of culture, the immunofluorescence assay was carried out based on staining for TRAP relying on ELF97 phosphatase substrate (Thermo Fisher Scientific), a fluorescent stain DAPI (4 ', 6-diamidino-2-phenylindole) was used for the detection of nuclei and phalloidin staining (Thermo Fisher Scientific) for acting ring. A Nikon microscope was used to highlight fluorescence using the appropriate filter and wavelength. Image processing was done using ImageJ software.

### Osteoassay

After 21 days of culture, the mature osteoclasts on the osteoassay plate were removed using 0.5% bleach followed by several washes. Bone resorption was assessed using a SMZ800 stereomicroscope Nikon microscope with a Nikon camera. Image processing was done using mosaicJ which is one of ImageJ software plugings*.*

### Quantitative real-time PCR

Both total RNA extraction and cDNA synthesis were performed as previously published [25]. Oligoprimer pairs for *DOCK2, DOCK5, DOCK6, ISG15, RAC1, SQSTM1*, and house keeping genes were designed by GeneTool 2.0 software (Biotools Inc, Edmonton, AB, CA) and their specificity was verified by blast in the GenBank database (Additional file 1: Table S1). The synthesis was performed by IDT (Integrated DNA Technology, Coralville, IA, USA). cDNA corresponding to 10–20 ng of total RNA was used to perform fluorescent-based Realtime PCR quantification using the LightCycler 480 (Roche Diagnostics, Mannheim, DE). Reagent LightCycler 480 SYBRGreen I Master (Roche Diagnostics, Indianapolis, IN, USA) was used as described by the manufacturer with 2% DMSO. The conditions for PCR reactions were: 45 cycles, denaturation at 98 °C for 10 s, annealing at 58 °C for 10 s, elongation at 72 °C for 14 s and then 74 °C for 5 s (reading). A melting curve was performed to assess non-specific signal. Calculation of the number of copies of each mRNA was performed according to Luu-The et al. [26] using second derivative method and a standard curve of Cp versus logarithm of the quantity. The standard curve was established using known amounts of purified PCR products (10, 102, 103, 104, 105 and 106 copies) and a LightCycler 480 v1.5 program provided by the manufacturer (Roche Diagnostics, Mannheim, DE). PCR amplification efficiency was verified. Normalization was performed using the reference genes shown to be genes having stable expression levels from embryonic life through adulthood in various tissues [27]: glucose-6-phosphate dehydrogenase (G6PD), peptidylprolyl isomerase B (cyclophilin B) (PPIB). Quantitative Real-Time PCR measurements were performed by the CHU de Québec Research Centre (CHUL) Gene Expression Platform, Quebec City, Canada and were compliant with MIQE guidelines [28, 29].

### Protein expression analyses by Western blot

The cell lysates were recovered in Laemmli buffer then the protein assay was carried out using the PierceTM 660 nm protocol. The reading was done with spectrophotometer using the TCAN program. We separated cell lysate using SDS-PAGE and transferred them to a PVDF membrane. The primary antibody incubation was done for 24 h against SQSTM1 (Cell signaling technology), DOCK6 (Proteintech, USA), RAC1 (Sigma Aldrich), DOCK2 (EMD Millipore), DOCK5 (LifeSpan BioSciences), anti-α-tubulin antibody (Cell signaling technology) and anti-vinculin antibody as controls. We used HRP-conjugated secondary anti-rabbit or anti-mouse antibodies (Cell signaling technology) for one-hour incubation to perform the detection with a chemiluminescent system. The protein expression quantification was done by the use of the Molecular Imager Gel Doc XR and Imaging System.

### Tridimensional models of SQSMT1 and DOCK6

These models were generated by AlphaFold 2 (and RoseTTAFold as a comparator to AlphaFold 2) software tools from their amino-acid sequences [30, 31]. AlphaFold 2, developed by Google AI offshoot DeepMind, uses artificial intelligence tools to predict, with high accuracy, the folding of proteins. RoseTTAFold uses deep learning under form of a “three-track” neural network, to accurately predict protein structures based on amino-acid sequence of query and aligned homologues sequences information. AlphaFold 2 has a per-residue confidence metric called predicted local distance difference test, which is used to colour the residues of the prediction. Thus, a predicted local distance difference test above 90 is of high confidence, between 90 and 70 of intermediate confidence, between 70 and 50 of low confidence, and the coordinates of any residue with a predicted local distance difference test below 50 should be questioned when interpreting structural features. It was found that the lower confidence regions are strongly correlated with disorder. Structural similarity of the obtained tridimensional (3D) models was performed with PDBeFold (https://www.ebi.ac.uk/msd-srv/ssm/ssmstart.html) against PDB database. We evaluated the potential impact of p.Pro392Leu and p.Val45Ile variants on the stability and dynamics of 3D model of SQSMT1 and DOCK6, respectively, with the DynaMut and DynaMut2 programs (http://biosig.unimelb.edu.au/dynamut/ and http://biosig.unimelb.edu.au/dynamut2) [32, 33]. DynaMut allows the analysis and visualization changes in protein stability and dynamics resulting from vibrational entropy and free energy changes caused by a mutation in protein structure using normal mode analysis. Structure images were generated using PyMOL (http://www.pymol.org).

### Statistical analyses

For the clinical phenotype analysis, we compared the age at diagnosis, the number of affected bones and the level of tALPs (expressed as the number of time the midpoint of normal range) in 14 patients within the two families carrying the p.Pro392Leu and/or p.Val45Ileu variants. For the assessment of the osteoclastic phenotype, we pooled patients with PDB or healthy carriers of the same mutation/variant to give more power to our statistical analysis. Ratio of multinucleated cells (actin positive cells with three nuclei or more over all actin positive cells, mean nuclei number per cell, and bone resorption abilities) were compared between each group. For gene and protein expression analyses, we presented the result as a ratio between the corresponding gene and protein expression versus the appropriate control. All statistical analyses were performed using GraphPad Prism, relying on ANOVA followed by Tukey post-tests. A p-value less than 0.05 was considered statistically significant.

## Results

### Whole exome sequencing

The filtering of whole exome sequencing raw data based on minor allele frequency below 1% retrieved 1,142 variants in family A and 1,141 variants in family B. Two hundred forty- two (242) rare variants in 191 different genes were shared by the four patients considered as phenocopies in both families. Interestingly, in silico prediction showed an interaction of 67 genes with the *SQSTM1* gene (Additional file 1: Table S2). Following intra-familial segregation analysis, review of the literature data and predicted gene and protein functions allowed us to select 106 candidate genes for the targeted sequencing. Among these 106 genes, 11 genes were part of the genes interacting in silico with the *SQSTM1* gene. The targeted sequencing and intra-family segregation analysis allowed us to identify 89 variants in 73 genes (Additional file 1: Table S3). Among these variants, six variants within five candidates’ genes were our best candidate of interest (Additional file 1: Table S4). Among these six variants, the variants in the *PEX5*, *TTN* and *MPRIP* genes were finally not confirmed by Sanger sequencing. The variant in the *TM4SF19* gene, initially kept in our selection considering its possible functional effect on osteoclast multinucleation as a member of the same family as DC-STAMP, segregated only within the family A. The p.Val45Ile rare variant in the *DOCK6* gene was finally our best candidate (rs183060698), based on its minor allele frequency, intra-familial segregation analysis as well as available data on prediction of gene and protein function. This variant was confirmed by Sanger sequencing in both families and displayed a high degree of conservation in evolution according to Homologene (Fig. 1B).

### Impact on the clinical phenotype of PDB

**Fig. 2:**
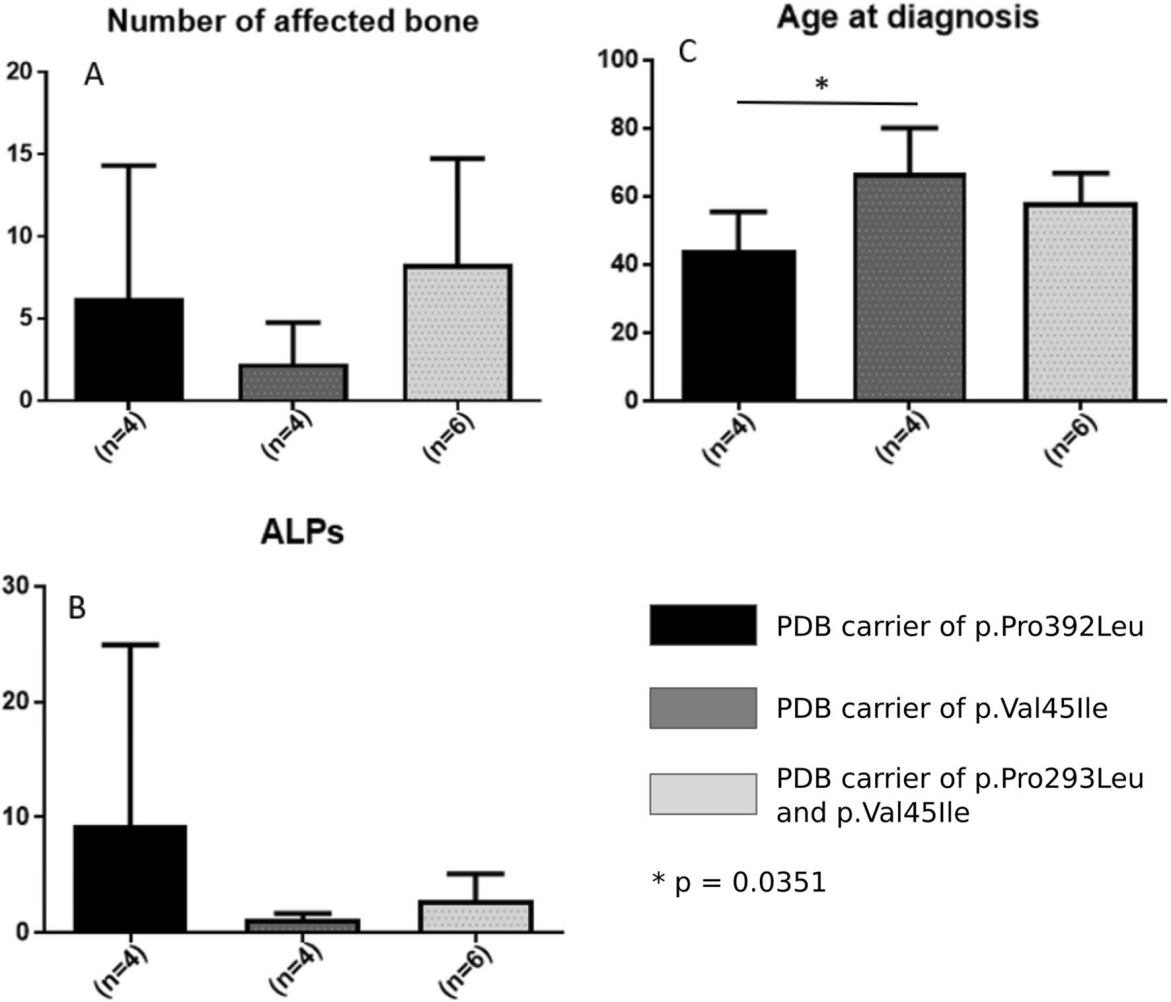
Impact on the clinical phenotype of Paget’s disease (PDB) of the *SQSTM1* and the *DOCK6* gene variants (number of affected bones, tALPs, age at PDB diagnosis). Data collection at baseline for forteen patients with PDB within the two families studied by whole exome sequencing. **A** Number of affected bones, **B** tALPs level, **C** age at diagnosis. *P = 0.035

We studied the clinical phenotype of PDB in 14 patients within the two families studied by whole exome sequencing. Among these 14 patients, 50% were men. The mean age at PDB diagnosis was significantly higher in patient carriers of the p.Val45Ile rare variant than in those carriers of the p.Pro392Leu mutation (67 vs. 44 years, *p* = 0.035) (Fig. 2).

### Impact on osteoclast morphology and bone resorption abilities

**Fig. 3:**
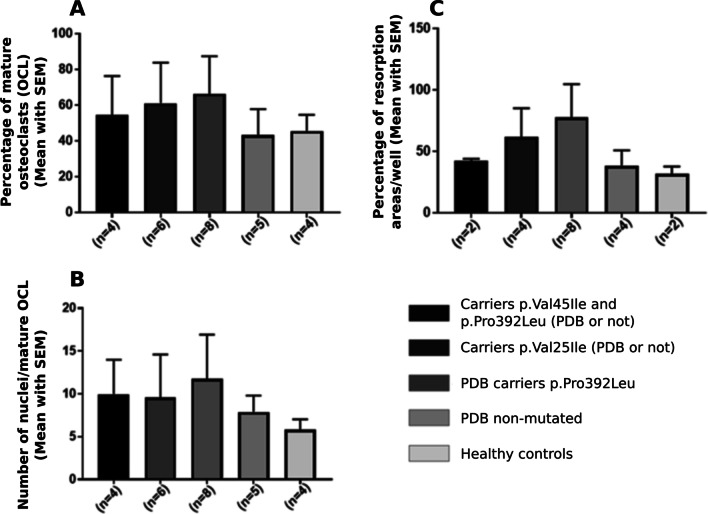
Impact on the osteoclastic phenotype of Paget’s disease (PDB) of the *SQSTM1* and *DOCK6* gene variants. Differentiation of PBMCs into mature osteoclasts (OCL) treated by RANKL and MCSF for 21 days. **A** Percentage of mature OCL. **B** Mean number of nuclei per mature OCL. **C** percentage of bone resorption areas

The osteoclast morphology and bone resorption abilities were studied in 27 participants. The osteoclastogenesis, the mean number of nuclei per cells, and the mean bone resorption were higher in patient carriers of the p.Pro392Leu mutation, in carriers of p.Val45Ile variant, in carriers of both variants than in healthy controls. Although the p.Val45Ile of the *DOCK6* gene and the p.Pro392Leu mutation both gave rise to a pagetic phenotype of osteoclast in comparison to healthy controls, this pagetic phenotype of osteoclasts was less severe in presence of the p.Val45Ile variant alone than in presence of the p.Pro392Leu mutation alone. The p.Val45Ile variant attenuated the severity of the osteoclastic phenotype caused by the p.Pro392Leu mutation when both variants were present (Fig. 3).

### mRNA expression analyses in osteoclasts

**Fig. 4:**
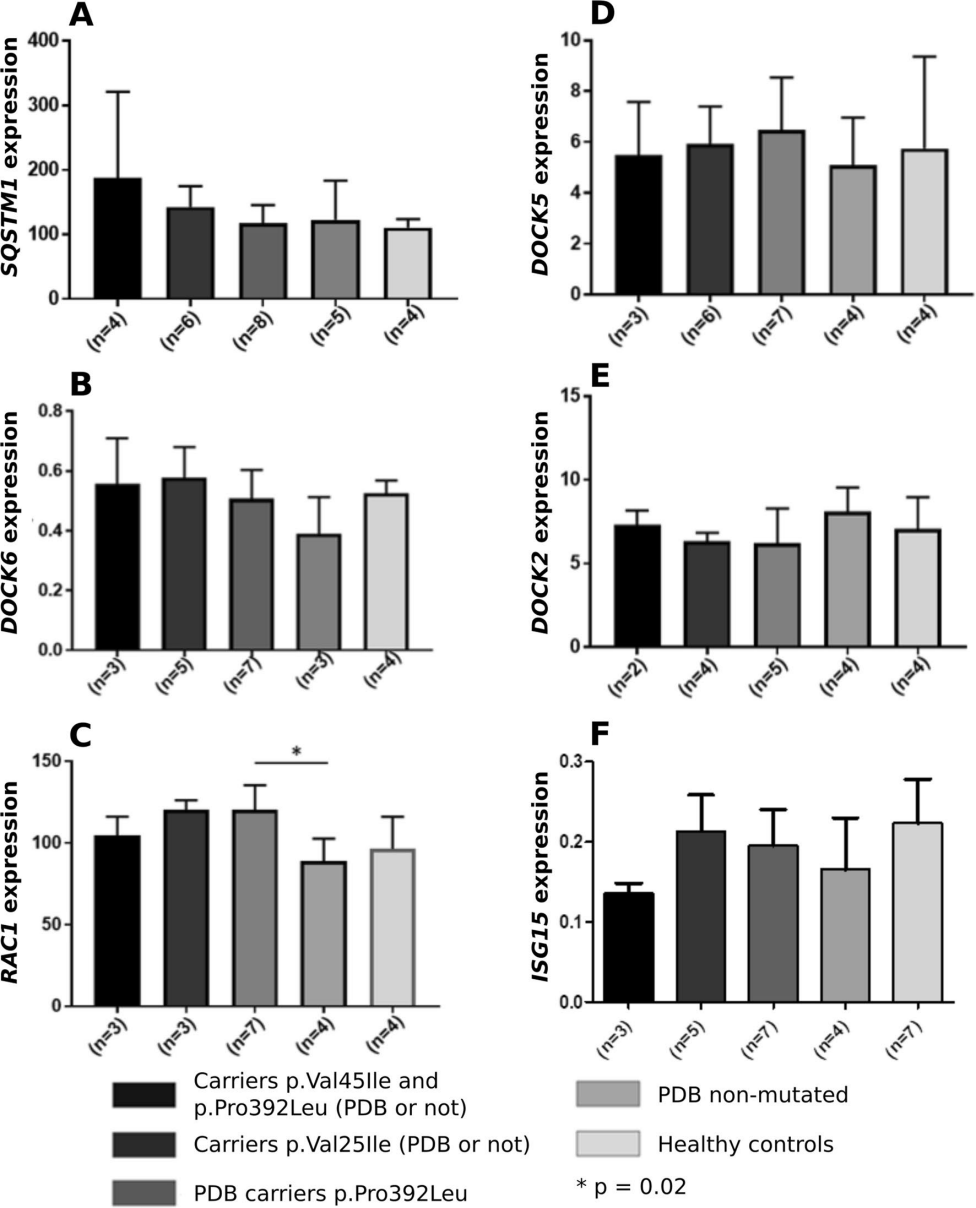
*SQSTM1*, *DOCKs*, *RAC1, ISG15* mRNA expressions in mature osteoclasts. *P = 0.02. **A** *SQSTM1* expression, **B** *DOCK6* expression, **C** *RAC1* expression, **D** *DOCK5* expression, **E** *DOCK2* expression, **F** *ISG15* expression

The *DOCK6* gene expression was higher in carriers of the p.Val45Ile variant than in other categories of patients or healthy controls (Fig. 4; Additional file 1: Table S5). The expression of the *RAC1* gene was increased in patient carriers of the p.Val45Ile variant and significantly higher in carriers of the p.Pro392Leu mutation versus patient not carriers of the mutation (120 vs. 89, *P* = 0.02). The *ubiquitin-like interferon (IFN)-stimulated gene 15 (ISG15)* gene expression was lower in carriers of both variants versus carriers of each variant separately.

### Protein expression analyses by Western Blot

**Fig. 5:**
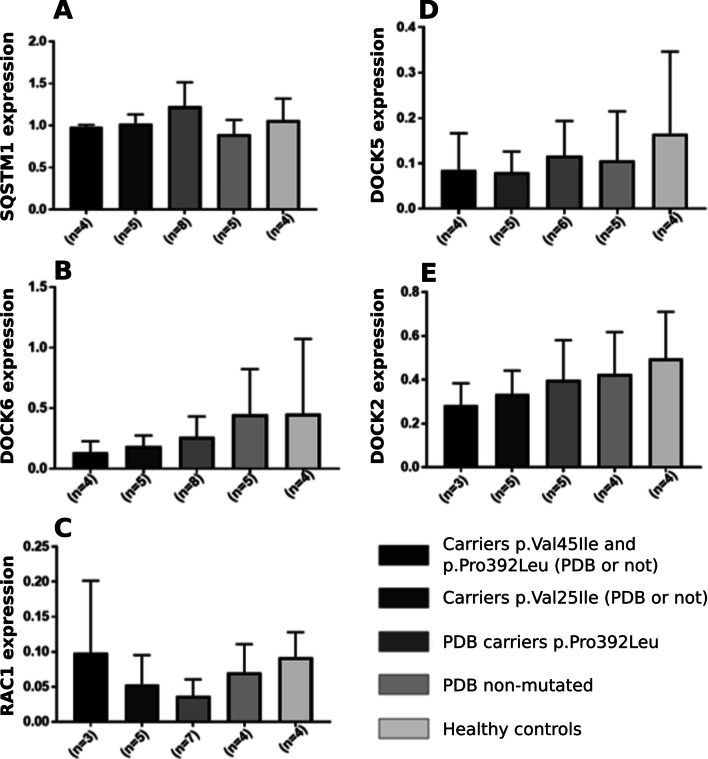
SQSTM1, DOCKs and RAC1 protein expressions in mature osteoclasts. **A** SQSTM1 expression, **B** DOCK6 expression, **C** RAC1 expression, **D** DOCK5 expression, **E** DOCK2 expression

The SQSTM1 protein expression was lower in carriers of the p.Val45Ile variant alone and in carriers of both variants than in patient carriers of the p.Pro392Leu mutation alone (Fig. 5; Additional file 1: Table S6). The DOCK6 protein expression was lower in carriers of the p.Val45Ile variant and in carriers of both variants than in patient carriers of the p.Pro392Leu mutation (Fig. 5). No significant changes were found in the expression of other proteins.

### Tridimensional model of SQSMT1

**Fig. 6:**
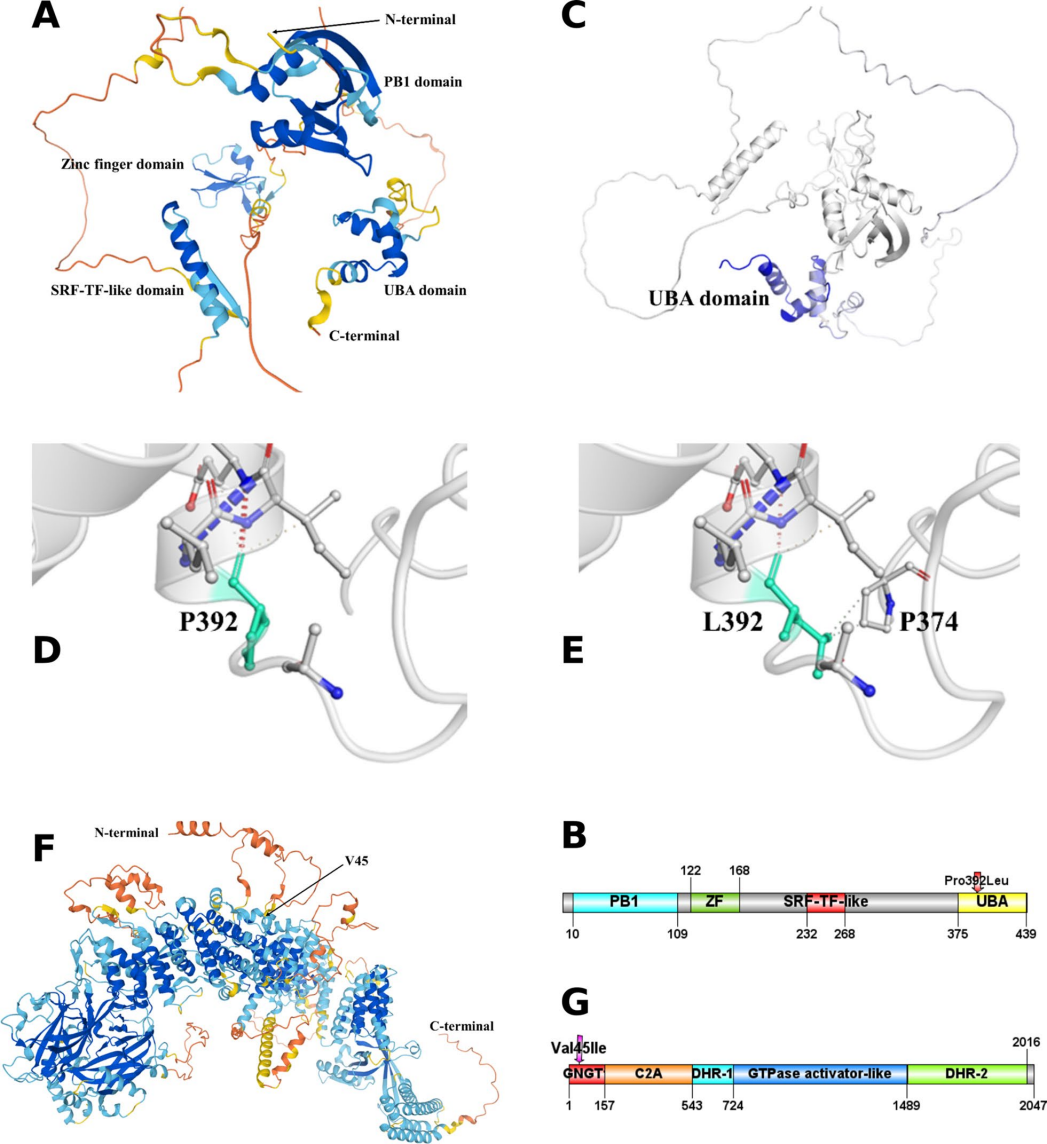
**A**, **B** 3D model of SQSTM1. AlphaFold 2 model for SQSTM1 (**A**). In addition to the structure of already known domains PB1 (amino acid 3–102), Zinc finger (122–168) and UBA (375–439), folding of SQSTM1 by AlphaFold 2 showed a new domain named Serum response factor-transcription factor (SRF-TF)-like (amino acid 232–268). AlphaFold 2 produced a per-residue confidence score (pLDDT) between 0 and 100: in blue, very high (pLDDT > 90); in cyan, confident (90 > pLDDT > 70); in yellow, low (70 > pLDDT > 50) and in orange, very low (pLDDT < 50). Some regions below 50 pLDDT may be unstructured in isolation. Diagram representation of SQSTM1 colored by domain (**B**) generated by http://ibs.biocuckoo.org/online.php#. **C**–**E** Prediction of the effect of the p.Pro392Leu mutation in stability and dynamics of SQSTM1 by DynaMut. The change in vibrational entropy energy of the UBA domain is colored based on the effect of the p.Pro392Leu mutation (**C**). The UBA domain is in blue that represents a rigidification of the structure relatively to the wild type. The predicted interatomic interactions for Pro392 (**D**) and Leu392 (**E**) residues are shown as sticks and colored in light green along with surrounding residues that are involved in any type of interactions. The p.Pro392Leu mutation created hydrophobic bonds (green dashes) between Leu392 and Pro374 (**E**). **F**, **G** 3D model of DOCK6. AlphaFold 2 model for DOCK6 (**F**). The p.Val45Ile variant is localized in N-terminal of DOCK6. This domain is mainly intrinsically disordered (regions in orange that are below 50 per-residue confidence score (pLDDT) may be unstructured in isolation). AlphaFold 2 produces a per-residue confidence score (pLDDT) between 0 and 100: in blue, very high (pLDDT > 90); in cyan, confident (90 > pLDDT > 70); in yellow, low (70 > pLDDT > 50) and in orange, very low (pLDDT < 50). Some regions below 50 pLDDT may be unstructured in isolation. Diagram representation of DOCK6 colored by domain (**G**) generated by http://ibs.biocuckoo.org/online.php#

The SQSTM1 protein folded by AlphaFold 2 presented three domains, which structure was already known: PB1 (amino acid 3–102), Zinc finger (122–168) and UBA (375–439), as well as a new domain (Fig. 6A, B). To determine its function, we used PDBeFold to search similar structures in PDB database. We found that it presents structural similarity with human serum response factor (SRF) (PDB id: 1K6O_C), DNA-binding domain of SEPALLATA 3 (PDB id: 7NB0_D) of *Arabidopsis thaliana* and MCM1 transcription factor (PDB id: 1MNM_B) of *Saccharomyces cerevisiae*. These transcription factors are Serum response factor-Transcription Factor (SRF-TF) (PFAM id: PF00319). Thus, we named the new domain (amino acid 232–268), the Serum response factor-Transcription Factor–like (SRF-TF-like) domain.

We evaluated the potential impact of the p.Pro392Leu mutation, localized in the UBA domain, on the stability and dynamics (protein motions) of SQSTM1 by analysing its 3D model (Fig. 6A, B) with DynaMut software. DynaMut predicted that this mutation has a stabilizing effect on the UBA structure associated with a decrease in flexibility of the whole UBA domain (Fig. 6C). Indeed, ΔΔG predictions, a measure of the change in monomeric protein stability when a point mutation is introduced, showed a stabilizing effect of the p.Pro392Leu mutation (ΔΔG: 1.693 kcal/mol), and ΔΔSVib (Δ vibrational entropy energy measures protein dynamics (flexibility) between wild-type and mutant) showed a decrease in molecule flexibility (ΔΔSVib ENCoM: − 1.337 kcal mol^−1^ K^−1^). These results are visualized in Fig. 6D, E where the p.Pro392Leu mutation created hydrophobic bonds between Leu392 and Pro374. Therefore, the p.Pro392Leu mutation may stabilize and reduce the flexibility of the UBA domain. This rigidity of the UBA domain may decrease possible interactions or crosstalk with the other SQSTM1 domains, mostly with the neighbor SRF-TF-like domain.

### Tridimensional model of DOCK6

3D model of DOCK6 (Fig. 6F, G) presents in addition to the two domains of already known structure, namely DHR-1 (amino acid 544–723) and DHR-2 (1490–2016), another new structural domains. Searching with PDBeFold software in PDB database revealed the putative function of these domains. Indeed, region between amino acid 1–157 (PFAM id: DUF3398) that contains the p.Val45Ile variant presented structural similarity with guanine nucleotide-binding protein G(T) subunit gamma-T1 (PDB id: 1TBG_H) of the heterotrimeric G protein complex (GNGT1, UniProt id: P02698). This complex contains a G protein gamma-subunit-like domain (PFAM id: PF00631), also found in regulators of G protein signaling proteins [34]. Region containing amino acids 158–543 has a fold similar to C2A domain (PFAM id: PF00168) of human Dysferlin (PDB id: 4IHB_E), which is involved in targeting proteins to cell membranes. This new domain revealed by AlphaFold 2 could explain why the DHR-1 domain (amino acid 544–723) alone is not sufficient to direct endoplasmic reticulum localization of DOCK6 [35]. Finally, amino acids 724 to 1489 correspond to the GTPase activator-like protein (PDB id: 5HIU_A). Interestingly, these new domains in DOCK6 have putative function related to guanine nucleotide exchange factors (GEFs).

**Fig. 7:**
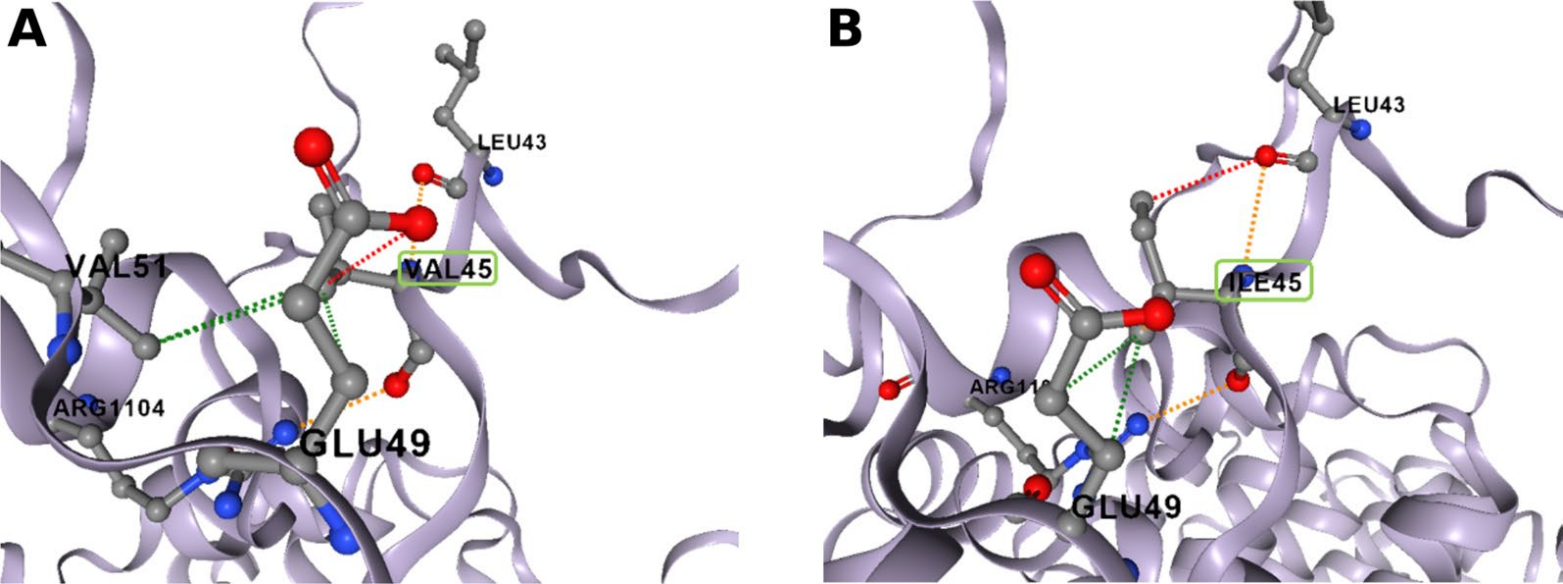
Prediction of the effect of the p.Val45Ile variant in stability and dynamics of DOCK6 by DynaMut2. The predicted interatomic interactions for Val45 (**A**) and Ile45 (**B**) residues are shown as sticks along with surrounding residues that are involved in any type of interactions. The p.Val45Ile variant mainly created hydrogen (red dashes) and polar (orange dashes) bonds between Ileu45 and Leu43 (**B**), and hydrophobic (green dashes) and polar (orange) bonds between Ileu45 and Glu49. In addition, the p.Val45Ile variant broke hydrophobic bonds between Val51 and Val45 (**A** comparatively to **B**). Val45 and Ile45 are boxed in green rectangle

We evaluated the potential impact of the p.Val45Ile variant on the stability and dynamics (protein motions) of DOCK6 by analysing its 3D model by DynaMut2 software. DynaMut2 predicted that this rare variant has a destabilizing effect on N-terminal structure associated with an increase in flexibility of the N-terminal extremity. Indeed, ΔΔG predictions, a measure of the change in monomeric protein stability when a point mutation is introduced, showed a destabilizing effect of the p.Val45Ile variant (ΔΔG: − 0.6 kcal/mol). These results are visualized in Fig. 7 where the p.Val45Ile variant mainly created hydrogen and polar bonds between Ileu45 and Leu43 and hydrophobic and polar bonds between Ileu45 and Glu49 (Fig. 7B). However, the p.Val45Ile variant also broke hydrophobic bonds between Val51 and Val45 (Fig. 7A comparatively to 7B). This destabilization of the region containing the putative G protein gamma-subunit-like domain could then alter the activity of DOCK6 on Rho-GTPases. Alteration of DOCK6 activity by the p.Val45Ile variant may increase levels of globular actin (G-actin) that sequester Myocardin-Related Transcription Factor A (MRTF-A), a co-activator of SRF-TF, leading to a decrease in SRF-TF transcription activity.

## Discussion

Whole exome sequencing in two large families in which patients with PDB carriers of the p.Pro392Leu mutation or not coexist in the same sibship allowed us to identify the p.Val45Ile rare variant in the *DOCK6* gene. In silico predictions for this variant vary from tolerated, benign or neutral to damaging and disease-causing according to MutationTaster. This rare missense variant is classified as a variant of uncertain significance with a minor allele frequency of 0.00176 according to Varsome database. In patients with PDB carriers of the p.Val45Ile rare variant, the age at PDB diagnosis was delayed versus patients with PDB carrier of the p.Pro392Leu mutation and the number of affected bones was lower. In addition, we observed a lower level of tALPs in patients with PDB carriers of the p.Val45Ile rare variant than in patients carrying the p.Pro392Leu mutation alone. The p.Val45Ile rare variant alone gives rise to a pagetic osteoclastic phenotype when compared to healthy controls, with increased osteoclastogenesis (61% vs. 45.5%), increased mean number of nuclei per osteoclast (9.5 vs. 5.8) and increased bone resorption area, (61.5% vs. 31.8%). This osteoclastic phenotype was less severe than the one observed in patient carriers of the p.Pro392Leu mutation alone. In carriers of both variants (patients with PDB or healthy carriers), the p.Val45Ile variant attenuated the severity of the cellular phenotype caused by the p.Pro392Leu mutation.

To better understand the impact of the missense rare variant p.Val45Ile on DOCK6 signaling, we looked at the gene and protein expression of RAC1 because DOCK6 is a GEF that exchanges GDP for GTP (activates) for RAC1 and CDC42. The RAC1 protein expression in carriers of both variants (patients with PDB or healthy carriers) was higher than in healthy controls whereas the DOCK6 protein expression was decreased. The osteoclasts carrying the *DOCK6* rare variant, either alone or in combination with the p.Pro392Leu mutation, had decreased levels of the DOCK6 protein in comparison to healthy controls, whereas the RAC1 protein expression was increased. Our results of the *ISG15* gene expression are also of interest, as ISG15 conjugation marks proteins for interaction with SQSTM1 when autophagy is stimulated [36]. Furthermore, according to the literature, a decrease in ISG15 levels suppresses the loss of DOCK6 function [35].

DOCK6 is a GEF that exchanges GDP for GTP (activates) for RAC1 and CDC42, which are small GTPases from the Rho family. The latter are critical regulators of the changes in the actin cytoskeleton [37]. In addition to actin cytoskeleton, Dock proteins also regulate cell adhesion and migration [38, 39]. DOCK6 contains three domains: DUF3398, the catalytic Dock Homology Region-2 (DHR-2) and DHR-1 [40, 41]. The DHR-1 domain of some DOCK-family proteins has been reported to be a novel type of phosphatidylinositol (PtdIns) [3–5] P3 (PIP3)-binding sequences [42]. Cerikan and Schiebel showed that DOCK6 localizes to the endoplasmic reticulum in dependence of its DHR-1 domain [35]. The DHR-2 domain catalyzes nucleotide exchange on Rac1 or Cdc42. The *DOCK6* gene expression is well described in neuronal differentiation, an increased expression of DOCK6 being necessary for neurite outgrowth [43]. Miyamoto et al. [40] suggested also that the effect of DOCK6 on axon growth is mediated via the activation of Rac1. Nevertheless, Cdc42 is required for axon growth and it is also a target of DOCK6 [44]. In humans, mutations in the *DOCK6* gene were linked to autosomal recessive forms of the Adams–Oliver syndrome-2 (AOS). Patients with this rare disease have hand and feet developmental defects associated to skin abnormalities [45, 46].

Rare variants of the *DOCK6* gene have never been reported in patients with bone disorders in the literature and its expression in osteoclasts is unknown. Among all DOCK family members, only DOCK5 expression was reported in osteoclasts in which it is strongly expressed and localizes to podosomes in the sealing zone [47, 48]. We determined that DOCK6 was expressed in human osteoclasts and may be involved in bone resorption.

Using last revolutionary AlphaFold 2 software, we described a new domain (amino acid 232–268) close to the UBA domain in SQSTM1, the SRF-TF-like domain. In mouse, SRF-TF binds to the serum response element of some genes. In complex with MRTF-A transcription coactivator, it controls expression of genes that regulate the cytoskeleton during development, morphogenesis and cell migration. The activity of SRF-TF-MRTF-A complex is sensitive to Rho GTPase-induced changes in G-actin concentration, thus coupling cytoskeletal gene expression to cytoskeletal dynamics [49]. SRF-TF is also involved in osteoblast differentiation and mineralization [50, 51]. The actin polymerization state can control genes transcription by SRF-TF-MRTF-A complex [52]. MRTF-A is a co-activator of SRF-TF. We showed that the p.Pro392Leu mutation might rigidify the UBA domain and thus decrease its possible interaction with SRF-TF-like domain. The decrease of this interaction may also alter the binding of MRTF-A to SRF-TF-like domain and thus decrease its transcription activity. Interestingly, Hocking et al. previously showed that the p.Pro392Leu mutation linked to PDB was not simply the result of the polyubiquitin binding properties of the mutant UBA domain, as the UBA domain could interact with protein(s) that are involved in bone cell function and that mutations in the UBA domain may impair this interaction [53].

Alteration of DOCK6 activity may increase levels of globular actin (G-actin) that sequester MRTF-A leading to a decrease in SRF-TF activity [54]. Therefore, both p.Pro392Leu mutation and p.Val45Ile variant have effect on SRF-TF. The p.Pro392Leu mutation contributes to the disturbance of possible interaction between the UBA domain and SRF-TF-like domain, while the p.Val45Ile variant contributes to the sequestration by G-actin of the MRTF-A, which is the co-activator of SRF-TF.

The main limitation of this study was the small number of participants in each step of our project, giving rise to descriptive results, as access to carriers of the *DOCK6* gene rare variant was limited due to its rarity. Overall, we found that the p.Val45Ile rare variant in the *DOCK6* gene may play a modifier role for the p.Pro392Leu mutation both at the clinical and osteoclastic levels. In our two families, a pseudo digenic pattern of inheritance remains then possible. Digenism is characterized by an inheritance of a single primary mutation that causes the disease, and a second genetic variant which modifies the clinical phenotype caused by the primary mutation [55]. The founder effect of the French-Canadian population may have contributed at this digenic inheritance in some pedigrees of our cohort. Further studies of the impact of this rare missense variant of the *DOCK6* gene on the actin cytoskeletal reorganization, on GTPases function, and on the SRF-TF-MRTF-A complex, represent interesting perspectives of this project.

## Conclusion

We identified a modifier effect of the p.Val45Ile rare variant in the *DOCK6* gene which attenuates the severity of the clinical phenotype of PDB linked to the p.Pro392Leu mutation, when both variants are carried by the same patient. According to our data, the p.Val45Ile rare variant gives rise to a pagetic osteoclastic phenotype, although less severe than the one observed with the p.Pro392Leu mutation alone.

## Supplementary Information

**Additional file 1.** Supplementary data.

## Data Availability

The p.Val45Ile variant in the *DOCK6* gene is already referenced as rs183060698 in public database. Raw data of the gene expression analyzes are available on Additional file 1: Table S5. Raw data of the protein expression analyzes are available on Additional file 1: Table S6. To preserve the privacy of research participants, the clinical datasets used and/or analysed during the current study are available from the corresponding author on reasonable request only.

## Abbreviations

PDB: Paget's disease of bone
UBA: Ubiquitin-associated domain
DC-STAMP: Dendritic cell-specific transmembrane protein
RANK: Receptor activator of nuclear factor κ B
MVNP: Measles virus nucleocapsids
GEF: Guanine nucleotide exchange factor protein
SRF-TF: Serum response factor–transcription factor domain
MRTF-A: Myocardin-related transcription factor A
