# Supplementary material for: Attenuated clinical and osteoclastic phenotypes of Paget’s disease of bone linked to the p.Pro392Leu/SQSTM1 mutation by a rare variant in the DOCK6 gene
Source: BMC Med Genomics. 2022 Mar 3;15:41. doi: 10.1186/s12920-022-01198-9 (PMC8895793; doi:10.1186/s12920-022-01198-9)

**Supplementary material**

**Supplementary table 1**: Sequence primers for gene expression quantification and gene description

| **Gene Symbol** | **Description** | **GenBank** | **Size (bp)** | **Primer sequence 5'→3'**  **F/R** |
| --- | --- | --- | --- | --- |
| ***HSD3B2*** | Homo sapiens 3-beta-hydroxysteroid dehydrogenase/delta-5-delta-4-isomerase (3-beta-HSD) gene (intron) | M38180 | 260 | GAAGGGCAGAGGTGGAACTAGAA/AACAAAGACCAAAGACCAGTGAGA |
| ***DOCK2*** | Homo sapiens dedicator of cytokinesis 2 (DOCK2) | NM_004946 | 201 | CCCACTCTCCATGCTCCTGAAC/GACACCCTTTTCTCATGGATCTTAAT |
| ***DOCK5*** | Homo sapiens dedicator of cytokinesis 5 (DOCK5) | NM_024940 | 130 | AAGCCGCACGGGGTCTATTGT/CGGTCTGGAAGGAGCATTGTCA |
| ***DOCK6*** | Homo sapiens dedicator of cytokinesis 6 (DOCK6) | NM_020812 | 146 | CGAGGACATGCAGAAGAAGACAC/CTAAAAACACCTGGGCCACCT |
| ***G6PD*** | Homo sapiens glucose-6-phosphate dehydrogenase (G6PD). nuclear gene encoding mitochondrial protein | NM_000402 | 121 | GATGTCCCCTGTCCCACCAACTCTG/GCAGGGCATTGAGGTTGGGAG |
| ***ISG15*** | Homo sapiens ISG15 ubiquitin like modifier (ISG15) | NM_005101 | 148 | CGACGAACCTCTGAGCATCCT/CTCGAAGGTCAGCCAGAACAG |
| ***PPIB*** | Homo sapiens peptidylprolyl isomerase B (cyclophilin B) (PPIB) | NM_000942 | 179 | GAAGAAGGGGCCCAAAGTCAC/CACGATGGAATTTGCTGTTTTTGTAG |
| ***RAC1*** | Homo sapiens Rac family small GTPase 1 (RAC1). 2 transcripts | NM_018890 | 180 | GACGGAGCTGTAGGTAAAACTTGC/TAGGGGGCGTAATCTGTCATAATCT |
| ***SQSTM1*** | Homo sapiens sequestosome 1 (SQSTM1). 3 transcripts | NM_003900 | 157 | GGCGGAGCAGATGAGGAAGAT/TGGCATCTGTAGGGACTGGAG |

**Supplementary table 2:** Genes in which rare variants were detected by the whole exome analysis and predicted *in silico* to interact with the *SQSTM1* gene.

| **Co-expression** | **Genetic interaction** | **Physical interaction** | **Shared protein domain** | **Colocalization** |
| --- | --- | --- | --- | --- |
| *AHNAK2* | *CCDC171* | *AIMP1* | *DMD* | *SLC29A2* |
| *ANKRD33* | *CCDC7* | *CD44* | *DTNB* | *TMEM38A* |
| *CD44* | *CNTNAP2* | *CHDH* | *UTRN* |  |
| *CEBPA* | *CTGF* | *CUL2* |  |  |
| *CHMP6* | *JADE1* | *DNAI2* |  |  |
| *CIC* | *MAP3K5* | *FLNB* |  |  |
| *DEDD* | *MEOX2* | *LLGL2* |  |  |
| *DPEP3* | *PCLO* | *LUC7L* |  |  |
| *FBN3* | *PDE7B* | *MCM5* |  |  |
| *FLNB* | *PGAM2* | *MLH1* |  |  |
| *HELZ2* | *RAPGEF1* | *MRPS9* |  |  |
| *HEXA* | *REXO2* | *MYO1E* |  |  |
| *HLA-A* | *RFX3* | *NDUFA5* |  |  |
| *IDH3A* | *TANK* | *PEX5** |  |  |
| *IFI35* | *THEMIS* | *RARS* |  |  |
| *IRF9* | *TNRC6C* | *SRRM2* |  |  |
| *LILRB4* | *TOR1AIP1* | *TRIB3* |  |  |
| *MAP2K1* | *TSPAN9* | *TRMT61B* |  |  |
| *NR4A2* |  | *ZBTB11* |  |  |
| *OXA1L* |  | *TTN** |  |  |
| *PLD2* |  |  |  |  |
| *SLC22A18AS* |  |  |  |  |
| *SRRM2* |  |  |  |  |
| *TANK* |  |  |  |  |
|  |  |  |  |  |
| *TCF25* |  |  |  |  |
| *TOR1AIP1* |  |  |  |  |
| *UGT2A1* |  |  |  |  |
| *WASF2* |  |  |  |  |
| *YIF1A* |  |  |  |  |

* Rare variants in these genes perfectly segregated with the clinical phenotype of PDB in the two families but were not further confirmed by Sanger sequencing.

**Supplementary table 3**: Variants detected in the targeted sequencing after intra-familial segregation analysis. AF-ALL: allele frequency from all populations. AF-AMR: allele frequency Mixed American population. AF-EUR: allele frequency European population.

| **Variant**  **name** | **Variant**  **Symbol symbol** | **Chromo-**  **Some** | **Position** | **Fonction** | **A F_**  **ALL** | **AF_**  **AMR** | **AF_**  **EUR** | **SIFT**  **_ pred** | **POlyph**  **en2_ HDIV_pred** |
| --- | --- | --- | --- | --- | --- | --- | --- | --- | --- |
| Neuron  Navigator 1 | NAV1 | 1 | 201772817 | May be involved in neuronal  Migration | 2.847e-05 | 0 | 0.000 | D | D |
| Ankyrin  repeat  domain 36 | ANKRD36 | 2 | 97862486 | Increased vaccinia virus (VACV)  infection | 0.0007 | 0.0003 | 0.0004560 | D | B |
| Crystallin  Gamma C | CRYGC | 2 | 208994187 | Dominant structural components  of the vertebrate eye lens | 0.0001 | 0.0001 | 0.0001641 | D | P |
| Striated  muscle  preferentially  expressed  Protein kinase | SPEG | 2 | 220342374 | May have a role in regulating the  growth and differentiation  of arterial smooth muscle cells | 0.0033 | 0.0010 | 0.001742 | NA | NA |
| Serine/  Threonine  Kinase 11  Interacting  Protein | STK11IP | 2 | 220472678 | May regulate STK11/LKB1 function  by controlling its subcellular  localization | NA | NA | NA | NA | NA |
| Transmembrane  4 L Six Family  Member 19 | TM4SF19 | 3 | 196050728 | Strongly decreased CFP-tsO45G  Cell surface transport  decreased homologous  recombination repair frequency | 0.0044 | 0.0028 | 0.006087 | D | B |
| Gamma-  A aminobutyric Acid  Type A Receptor  Alpha2 Subunit | GABRA2 | 4 | 46312266 | Major inhibitory neurotransmitter  in the vertebrate brain | 1.22e-05 | 0 | 0.000 | NA | NA |
| Sperenesis matog  Associated  18 | SPATA18 | 4 | 52943061 | Key regulator of mitochondrial  quality that mediates the  repairing or degradation of unhealthy mitochondria in response  to mitochondrial damage | 0.0074 | 0.0039 | 0.001672 | T | B |
| UDP  Glucuronosyl-  transferase  Fam2 Member  A1  Complex Locus | UGT2A1 | 4 | 70513199 | Catalyze phase II biotransformation reactions in which lipophilic substrates are  conjugated with glucuronic  acid to increase water solubility  and enhance excretion | 0.0067 | 0.0051 | 0.008358 | D | D |
| Aryl-  hydrocarbon  Receptor  repressor | AHRR | 5 | 434546 | Mediates dioxin toxicity and  is involve in in regulation  of cell growth and  differentiation | 0.0035 | 0.0011 | 0.008977 | T | B |
| Lysophosph-  atidyl-  choline  Acyltrans-  ferase | LPCAT1 | 5 | 1463932 | Possesses both acyltransferase  and acetyltransferase activities | 0.0009 | 0.0012 | 0.00007964 | D | B |
| Thymocyte  Selection  Associated | THEMIS | 6 | 128134106 | Plays a central role in late thymocyte development by controlling both positive and  negative T-cell selection | NA | NA | NA | D | B |
| TATA-Box  Binding  Protein Like 1 | TBPL1 | 6 | 134303785 | Part of a specialized transcription  system that mediates the  transcription of most ribosomal  proteins through the 5-TCT-3  motif which is a core promoter  element at these genes | 0.0018 | 0.0012 | 0.003227 | T | B |
| Mitogen-  Activated  Protein Kinase  Kinase Kinase 5 | MAP3K5 | 6 | 136882715 | Acts as an essential component of  the MAP kinase signal  transduction pathway | 0.0064 | 0.0038 | 0.0008359 | T | B |
| Mitogen-  Activated  Protein Kinase  Kinase Kinase 5 | MAP3K5 | 6 | 136926509 | Acts as an essential component  of the MAP kinase signal  transduction pathway | 0.0003 | 0.0018 | 0.000 | NA | NA |
| NHS Like 1 | NHSL1 | 6 | 138752241 | Motor neuron migration | 0.0008 | 8. 349e-05 | 0.000 | D | D |
| Spectrin Repeat  Containing Nuclear Envelope  Protein 1 | SYNE1 | 6 | 152462200 | Multi-isomeric modular protein  which forms a linking network  between organelles and the  actin cytoskeleton to maintain  the subcellular  spatial organization | NA | NA | NA | NA | NA |
| Spectrin Repeat  Containing  Nuclear  Envelope  Protein 1 | SYNE1 | 6 | 152668215 | Multi-isomeric modular protein  which forms a linking network  between organelles and the  actin cytoskeleton to maintain  the subcellular  spatial organization | 0.0089 | 0 .0064 | 0.01047 | NA | NA |
| Spectrin Repeat  Containing  Nuclear  Envelope  Protein 1 | SYNE1 | 6 | 152690199 | Multi-isomeric modular protein  which forms a linking network  between organelles and the  actin cytoskeleton to maintain  the subcellular spatial  organization | 0.0007 | 0.0012 | 0.000 | T | P |
| Trinucleotide  Repeat  Containing 18 | TNRC18 | 7 | 5428706 | Chromatin binding. transcription regulatory region sequence-specific DNA binding | 0.0002 | 0 | 0.0001999 | D | D |
| NADH  Dehydro-  genase  [ubiquinone]  1 alpha  subcomplex  subunit 5 | NDUFA5 | 7 | 123197843 | NADH dehydrogenase  (ubiquinone) activity.  mitochondrial electron transport.  NADH to ubiquinone.  mitochondrial respiratory chain  complex I assembly | 0.0002 | 0 | 0.000 | D | D |
| Coiled-Coil  Domain  Containing  136 | CCDC136 | 7 | 128449696 | May play a role in acrosome  formation in spermatogenesis  and in fertilization | 0.0024 | 0.0041 | 0.00005570 | D | D |
| Lysine  Demethylase  7A | KDM7A | 7 | 139791645 | Histone demethylase required  for brain development | 0.0038 | 0.0008 | 0.0005199 | D | B |
| Kell  metallo-  endopeptidase | KEL | 7 | 142641809 | Zinc endopeptidase with  endothelin-3-converting enzyme  activity | 9.753e-05 | 0 | 0.000 | T | B |
| [SCO-Spondin](http://www.genenames.org/cgi-bin/gene_symbol_report?hgnc_id=21998) | SSPO | 7 | 149474392 | Involved in the modulation of  neuronal aggregation | 0.0002 | 0.0002 | 0.00005441 | . | B |
| [SCO-Spondin](http://www.genenames.org/cgi-bin/gene_symbol_report?hgnc_id=21998) | SSPO | 7 | 149485018 | Involved in the modulation of  neuronal aggregation | 0.0093 | 0.0042 | 0.002644 | . | P |
| [SCO-Spondin](http://www.genenames.org/cgi-bin/gene_symbol_report?hgnc_id=21998) | SSPO | 7 | 149505703 | Involved in the modulation of  neuronal aggregation | 0.0056 | 0.0034 | 0.001351 | . | B |
| Sonic  Hedgehog | SHH | 7 | 155595719 | Intercellular signal essential for a  variety of patterning events  during development | NA | NA | NA | D | P |
| Myomesin 2 | MYOM2 | 8 | 2092616 | Major component of the  vertebrate myofibrillar M band | NA | NA | NA | D | D |
| Fucosyl  transferase  10 | FUT10 | 8 | 33246689 | Probable fucosyltransferase | 5.289e-05 | 0.0003 | 0.000 | T | B |
| [Transmembrane Protein 261](http://www.genenames.org/cgi-bin/gene_symbol_report?hgnc_id=30536) | T MEM261 | 9 | 7799581 | Increased vaccinia virus (VACV)  infection | 3.662e-05 | 0 | 0.000 | T | B |
| Glucosaminyl  (N-Acetyl)  Transferase 1.  Core 2 | GCNT1 | 9 | 79118400 | Catalyzes the transfer of an  N-acetylglucosamine moiety  onto mucin-type core 1 O-glycan  to form the branched  mucin-type core 2 O-glycan | 0.0054 | 0.0033 | 0.002309 | D | D |
| Nuclear  Apoptosis  Inducing  Factor 1 | NAIF1 | 9 | 130829254 | Leukocyte extravasation as  they serve as scaffolds for the  display of the selectin ligand  sialyl lewis X by leucocyte | 0.0029 | 0.0019 | 0.0004038 | T | B |
| Cilia and  flagella  associated  protein 46 | CFAP46 | 10 | 134699391 | As part of the central apparatus  of the cilium axoneme plays a role  in cilium movement | 0.0045 | 0.0008 | 0.01079 | D 0 | ……. . |
| Cilia and  flagella  associated  protein 46 | CFAP46 | 10 | 134723673 | As part of the central apparatus  of the cilium axoneme plays a role  in cilium movement | NA | NA | NA | NA | NA |
| Plakophilin 3 | PKP3 | 11 | 403636 | May play a role in junctional  plaques | 3.338e-05 | 2. 983e-05 | 0.000 | D | B |
| Leucine Rich Repeat Containing 56 | LRRC56 | 11 | 551708 | Increased vaccinia  Virus (VACV) infection  Increased transferrin  (TF) endocytosis | 0.0052 | 0.0020 | 0.005533 | T | D |
| Lamin Tail  Domain  Containing 2 | LMNTD2 | 11 | 556049 | Decreased Hepatitis  C virus replication | 0.0051 | 0.0020 | 0.005446 | T | B |
| PHD And Ring  Finger  Domains 1 | PHRF1 | 11 | 607680 | Increased vaccinia  virus (VACV) infection | 0.0052 | 0.0021 | 0.006096 | D | B |
| T-Complex 11  Like 1 | TCP11L1 | 11 | 33094215 | Increased gamma-  H2AX phosphorylation | 0.0092 | 0.0029 | 0.01597 | T | B |
| Ubiquitin  Specific  Peptidase  Like 1 | USPL1 | 13 | 31232469 | SUMO-specific isopeptidase  involved in protein desumoylation | 4.492e-05 | 0.000 | 0.000 | T | D |
| [Periostin](http://www.genenames.org/cgi-bin/gene_symbol_report?hgnc_id=16953) | POSTN | 13 | 38143472 | Induces cell attachment  and spreading and plays a  role in cell adhesion | 0.0077 | 0.0081 | 0.002828 | T | B |
| [Periostin](http://www.genenames.org/cgi-bin/gene_symbol_report?hgnc_id=16953) | POSTN | 13 | 38164572 | Induces cell attachment and  spreading and plays a role in  cell adhesion | 1.638e-05 | 0.000 | 0.000 | NA | NA |
| OXA1L.  Mitochondrial  Inner  Membrane  Protein | OXA1L | 14 | 23235822 | Required for the insertion  of integral membrane proteins  into the mitochondrial inner  membrane | 0.0026 | 0.0005 | 0.002429 | T | P |
| Solute Carrier  Family 7  Member 7 | SLC7A7 | 14 | 23243191 | Involved in the sodium-  Independent uptake of dibasic  amino acids and sodium-  dependent uptake of some  neutral amino acids | 0.0023 | 0.0005 | 0.002429 | T | B |
| Solute Carrier  Family 7  Member 7 | SLC7A7 | 14 | 23243344 | Involved in the sodium-  independent uptake of dibasic  amino acids and sodium-  dependent uptake of some  neutral amino acids | 0.0005 | 0.000 | 0.000 | NA | NA |
| BUB1B-  PAK6  Readthrough | BUB1B-PAK6;PAK6 | 15 | 40564441 | Increased Vaccinia virus (VACV)  infection  shRNA abundance <= 50% | 0.0007 | 0.0003 | 0.001847 | T | B |
| RNA  Polymerase II  Associated  Protein 1 | RPAP1 | 15 | 41819466 | Forms an interface between  the RNA polymerase II enzyme  and chaperone/scaffolding  protein | 0.0051 | 0.0017 | 0.01348 | D | D |
| S PG11. Spatacsin  Vesicle  Trafficking  Associated | SPG11 | 15 | 44865000 | May play a role in neurite  plasticity by maintaining  cytoskeleton stability  and regulating synaptic  vesicle transport | 0.0030 | 0.0049 | 0.0003583 | T | B |
| Protein  Disulfide  Isomerase  Family A  Member 2 | PDIA2 | 16 | 334920 | Acts as an intracellular  estrogen-  binding protein | 0.0088 | 0.0089 | 0.003556 | . | . |
| Protein  Disulfide  Isomerase  Family A  Member 2 | PDIA2 | 16 | 335639 | Acts as an intracellular  estrogen-  binding protein | 0.0070 | 0.0065 | 0.001438 | T | B |
| Protein  Disulfide  Isomerase  Family A  Member 2 | PDIA2 | 16 | 336854 | Acts as an intracellular estrogen-  binding protein | 0.0046 | 0.0023 | 0.0004893 | T | B |
| Trinucleotide  Repeat  Containing 6A | TNRC6A | 16 | 24804800 | Plays a role in RNA-mediated gene silencing by both micro-RNAs (miRNAs)  And short  interfering RNAs (siRNAs) | 5.496e-05 | 0.000 | 0.000 | D | B |
| Myosin light  chain.  phosphory-  latable.  fast skeletal  muscle | MYLPF | 16 | 30388013 | Increased vaccinia virus  (VACV)  infection Increased  gamma-H2AX phosphorylation | 0.0004 | 0.0002 | 0.000 | D | B |
| Lon  Peptidase 2.  Peroxisomal | LONP2 | 16 | 48278430 | ATP-dependent serine protease  that mediates the selective  degradation of misfolded  and unassembled  polypeptides in the peroxisomal  matrix | 3.545e-05 | 0.000 | 0.000 | T | B |
| Pleckstrin  Homology  And RhoGEF  Domain  Containing G4 | PLEKHG4 | 16 | 67318670 | Possible role in intracellular  signaling and cytoskeleton  dynamics at the Golgi | 4.5e-05 | 0 | 0.000 | T | B |
| Centrobin.  Centriole  Duplication  And Spindle  Assembly  Protein | CNTROB | 17 | 7838453 | Required for centriole  duplication | 0.0015 | 0.0018 | 0.001157 | T | D |
| leucine rich  repeat  containing  37 member  A3 | LRRC37A3 | 17 | 62915559 | Increased viability Mildly  decreased  CFP-tsO45G cell surface transport | 0.0051 | 0.0052 | 0.009745 | . | . |
| Solute carrier  family 25  member 52 | SLC25  A52 | 18 | 29340258 | Increased vaccinia virus  (VACV) infection | 8.125e-06 | 0.000 | 0.000 | T | B |
| Melanocortin  4 Receptor | MC4R | 18 | 58038832 | Receptor specific to the  heptapeptide core common  to adrenocorticotropic hormone  and alpha-. beta-.  and gamma-MSH | 0.0069 | 0.0041 | 0.009752 | T | B |
| Ribosome  Binding  Factor A  (Putative) | RBFA | 18 | 77805778 | Increased viability  I Increased vaccinia  virus (VACV) infection | 0.0097 | 0 .0027 | 0.01377 | T | B |
| Dedicator  Of  Cytokinesis 6 | DOCK6 | 19 | 11326585 | May regulate neurite outgrowth | 0.0086 | 0.0028 | 0.02366 | D | B |
| Dedicator  Of  Cytokinesis 6 | DOCK6 | 19 | 11363634 | May regulate neurite outgrowth | 0.0017 | 0.0010 | 0.0003001 | T | B |
| Zinc Finger  Protein 440 | ZNF440 | 19 | 11942721 | May be involved in  transcriptional regulation | 0.0005 | 0.0003 | 0.00007980 | . | . |
| C3 And PZP  Like.  Alpha-2-  Macroglobulin  Domain  Containing 8 | CPAMD8 | 19 | 17104239 | Up-regulated by IL1B/  interleukin-1  beta and IL6/interleukin-6 | 0.0099 | 0.0062 | 0.003686 | T | P |
| SURP And  G-Patch  Domain  Containing 1 | SUGP1 | 19 | 19387869 | Plays a role in pre-mRNA splicing | 4.155e-06 | 0.000 | 0.000 | T | B |
| [WD 62](http://www.genenames.org/cgi-bin/gene_symbol_report?hgnc_id=24502)  Repeat  Domain | WDR62 | 19 | 36549757 | Cerebral cortical development.  neuronal proliferation  and migration. mother-centriole-  dependent centriole  duplication | 0.0012 | 0.0002 | 0.001235 | D | D |
| Seryl- TRNA  Synthetase 2.  Mitochondrial | SARS2 | 19 | 39421129 | Catalyzes the attachment  of serine to tRNA(Ser) | 0.0095 | 0.0073 | 0.004216 | T | P |
| Mitochondrial  Ribosomal  Protein S12 | MRPS12 | 19 | 39423329 | Increased vaccinia virus  (VACV) infection | 0.0049 | 0.0038 | 0.01947 | NA | NA |
| F-Box  Protein 17 | FBXO17 | 19 | 39435757 | Substrate-recognition component  of the SCF (SKP1-CUL1-F-  box protein  )-type E3 ubiquitin  ligase complex | 4.081e-06 | 0.000 | 0.000 | NA | NA |
| Zinc Finger  Protein 546 | ZNF546 | 19 | 40520798 | May be involved in  transcriptional regulation | 0.0022 | 0.0034 | 0.001355 | NA | NA |
| Glutamate  Receptor.  Ionotropic.  N-Methyl  D-Aspartate  2D | GRIN2D | 19 | 48917810 | Extracellular-glutamate-gated  ion channel activity | 6.53e-06 | 0.000 | 0.000 | D | D |
| Zinc Finger  Protein 610 | ZNF610 | 19 | 52857105 | May be involved in  transcriptional regulation | 0.0077 | 0.0045 | 0.0003029 | NA | NA |
| Zinc Finger  Protein 610 | ZNF610 | 19 | 52869431 | May be involved in  transcriptional regulation | 0.0013 | 0.0031 | 0.00007972 | D | D |
| Zinc Finger  Protein 610 | ZNF610 | 19 | 52869648 | May be involved in  transcriptional regulation | 0.0070 | 0.0033 | 0.00007976 | NA | NA |
| Zinc Finger  Protein 610 | ZNF610 | 19 | 52869997 | May be involved in  transcriptional regulation | 0.0071 | 0.0033 | 0.00007985 | NA | NA |
| Leucine  Zipper Tumor  Suppressor  Family  Member 3 | LZTS3 | 20 | 3146711 | Mitotic spindle defects  Increased vaccinia virus (VACV)  infection | 6.12e-05 | 8. 948e-05 | 0.00004003 | T | D |
| Sialic Acid  Binding  Ig Like  Lectin 1 | SIGLEC1 | 20 | 3669698 | Acts as an endocytic receptor  mediating clathrin dependent  endocytosis | NA | NA | NA | NA | NA |
| Potassium  Voltage-Gated  Channel  Subfamily E | KCNE1;  KCNE1B | 21 | 35821680 | Ancillary protein that assembles  as a beta subunit with a voltage-  gated potassium channel  complex of pore-forming alpha  subunits | 0.0095 | 0.0027 | 0.01688 | D | P |
| Trefoil  Factor 3 | TFF3 | 21 | 43733628 | Involved in the maintenance  and repair of the intestinal  mucosa | 0.0043 | 0.0032 | 0.001514 | D | D |
| Trefoil  Factor 2 | TFF2 | 21 | 43771047 | Inhibits gastrointestinal  motility and gastric acid secretion | 0.0058 | 0.0032 | 0.003544 | T | B |
| Sushi  Domain  Containing 2 | SUSD2 | 22 | 24579455 | May be a cytokine receptor for  C10ORF99 | 2.467e-05 | 0.000 | 0.000 | NA | NA |
| Sushi  Domain  Containing 2 | SUSD2 | 22 | 24583456 | May be a cytokine receptor for  C10ORF99 | 0.0008 | 0.0009 | 0.0001647 | NA | NA |
| Sushi  Domain  Containing 2 | SUSD2 | 22 | 24583953 | May be a cytokine receptor for  C10ORF99 | 0.0006 | 0.0009 | 0.0001594 | D | D |
| Myotubularin  Related  Protein 3 | MTMR3 | 22 | 30415983 | Phosphatase that acts on lipids  with a phosphoinositol  headgroup | 0.0059 | 0.0036 | 0.001871 | T | P |
| HORMA  Domain  Containing 2 | HORMAD2 | 22 | 30489945 | Essential for synapsis surveillance  during meiotic prophase  via the recruitment of ATR activity | 0.0057 | 0.0034 | 0.001805 | D | B |
| Myosin  Heavy  Chain 9 | MYH9 | 22 | 36745146 | Cellular myosin that appears to  play a role in cytokinesis. cell  shape. and specialized functions  such as secretion and capping | 0.0062 | 0.0005 | 0.04428 | D | P |
| Pyridoxal  Phosphatase | PDXP | 22 | 38061576 | Protein serine phosphatase that  dephosphorylates Ser-3 in cofilin  and probably alsohosphorylates  dep phospho-serine residues in  DSTN | 0.0011 | 0.0004 | 0.001667 | D | B |
| TRNA 5-  Methylamin-  omethyl-2-  Thiouridylate | TRMU | 22 | 46742350 | Catalyzes the 2-thiolation  of uridine  at the wobble position (U34)  of mitochondrial tRNA(Lys).  tRNA(Glu) and tRNA(Gln) | 0.0038 | 0.0033 | 0.002587 | NA | NA |

**Supplementary table 4:** Rare variants detected in the whole exome analysis and in the targeted sequencing which segregated within the two families.

| **Gene Symbol** | ***TM4SF19*** | ***PEX5*** | ***TTN*** | ***TTN*** | ***DOCK6*** | ***MPRIP*** |
| --- | --- | --- | --- | --- | --- | --- |
| **Gene name** | Transmembrane 4 L Six Family Member 19# | Peroxisomal Biogenesis Factor 5* | Titin* | Titin* | Dedicator Of Cytokinesis 6 | Myosin Phosphatase Rho Interacting Protein* |
| **Locus** | *3q29* | *12p13.31* | *2q31.2* | *2q31.2* | *19p13.2* | *17p11.2* |
| **Variant** | *T/C* | *GCCTCTGAG*  *GCAGTGAGT*  *GTTCTTGAG*  *GTGGAAAGC*  *CCAGGTGCA/-* | *G/A* | *G/A* | *C/T* | *CAG/-* |
| **Amino Acid** | p.Asn197Ser | p.192+32_192+76 | p.Arg32748Cys | p.His10092Tyr | p.Val45Ile | p.178_179 del |
| **Type of variation** | Substitution | Deletion | Substitution | Substitution | Substitution | Deletion |
| **Effect prediction**  **(SIFT)** | Tolerated | Not applicable | Damaging | Tolerated | Tolerated | Not applicable |
| **Effect prediction (PolyPhen 2)** | Benign | Not applicable | Probably  damaging | Benign | Benign | Not applicable |
| **Effect prediction (LTR)** | Neutral | Not applicable | Not applicable | Not applicable | Neutral | Not applicable |
| **Conservation during evolution** | YES | YES | YES | YES | YES | YES |
| **Function** | Osteoclast fusion by similarity | Autophagy | -Assembly of muscle  -Sarcomas | -Assembly of muscle  -Sarcomas | Proliferation and cell migration | Proliferation and cell migration |

# This rare variant segregated only within one family. * The rare variants of these genes were not further confirmed by Sanger Sequencing.

**Supplementary table 5:** Raw data of the gene expression analyses in osteoclasts

|  |  |  | **Cq (10-20 ng/rxn)** | | | | | |  |  |  |  |  |  |  |  |
| --- | --- | --- | --- | --- | --- | --- | --- | --- | --- | --- | --- | --- | --- | --- | --- | --- |
|  |  |  | **gDNA** | **Genes** | | **G6PD** | **PPIB** | **18S** | **number of copies/ug of total RNA** | | | | | | | |
| **#** | **Identification** | **Gene Symbol** |  | **Cq 1** | **Cq 2** | **Cq Mean** | **Cq Mean** | **Cq Mean** | **Q1** | **Q2** | **Mean** | **Standard deviation** | **G6PD** | **PPIB** | **18S** | **Geomean HK** |
| 1 | **Carriers p.Val45Ile and p.Pro392Leu (PDB or not)** | **SQSTM1** | 39.34 | 20.09 | 19.80 | 22.50 | 21.87 | 6.00 | 3 687 192 | 4 484 954 | **4 086 073** | 564 103 | 902 335 | 1 383 591 | 60 128 870 627 | 42 184 503 |
| 2 | **Healthy controls** | **SQSTM1** | 45.00 | 19.56 | 19.60 | 21.57 | 19.03 | 6.00 | 2 712 807 | 2 640 565 | **2 676 686** | 51 083 | 857 659 | 4 741 051 | 30 559 614 248 | 49 901 368 |
| 3 | **Carriers p.Val45Ile (PDB or not)** | **SQSTM1** | 45.00 | 21.01 | 20.90 | 21.29 | 22.41 | 6.00 | 1 672 724 | 1 801 752 | **1 737 238** | 91 236 | 1 724 905 | 811 591 | 50 902 747 621 | 41 458 593 |
| 4 | **Carriers p.Val45Ile and p.Pro392Leu (PDB or not)** | **SQSTM1** | 34.06 | 20.46 | 20.24 | 21.17 | 20.08 | 6.00 | 1 464 300 | 1 698 810 | **1 581 555** | 165 824 | 1 126 458 | 2 346 241 | 30 559 614 248 | 43 226 018 |
| 5 | **Carriers p.Val45Ile and p.Pro392Leu (PDB or not)** | **SQSTM1** | 34.66 | 18.47 | 18.60 | 19.08 | 19.12 | 6.00 | 5 661 436 | 5 185 975 | **5 423 706** | 336 202 | 4 584 134 | 4 477 393 | 30 559 614 248 | 85 600 598 |
| 6 | **Carriers p.Val45Ile and p.Pro392Leu (PDB or not)** | **SQSTM1** | 36.77 | 18.15 | 17.87 | 18.73 | 19.57 | 6.00 | 7 146 803 | 8 630 797 | **7 888 800** | 1 049 342 | 5 821 572 | 3 296 155 | 30 559 614 248 | 83 701 245 |
| 7 | **Carriers p.Val45Ile (PDB or not)** | **SQSTM1** | 34.95 | 18.62 | 18.66 | 19.08 | 19.69 | 6.00 | 5 008 588 | 4 874 988 | **4 941 788** | 94 470 | 4 584 134 | 3 050 618 | 30 559 614 248 | 75 323 586 |
| 8 | **Carriers p.Val45Ile (PDB or not)** | **SQSTM1** | 33.55 | 18.30 | 18.18 | 18.49 | 19.63 | 6.00 | 6 191 018 | 6 714 286 | **6 452 652** | 370 006 | 6 842 321 | 3 176 351 | 30 559 614 248 | 87 248 897 |
| 9 | **Carriers p.Val45Ile (PDB or not)** | **SQSTM1** | 33.16 | 18.76 | 18.68 | 18.70 | 19.20 | 6.00 | 4 502 631 | 4 753 045 | **4 627 838** | 177 069 | 5 940 331 | 4 228 397 | 30 559 614 248 | 91 561 191 |
| 10 | **Carriers p.Val45Ile (PDB or not)** | **SQSTM1** | 34.37 | 18.29 | 18.24 | 19.50 | 19.37 | 6.00 | 6 398 655 | 6 618 201 | **6 508 428** | 155 242 | 3 466 838 | 3 783 891 | 30 559 614 248 | 73 734 943 |
| 11 | **Carriers p.Val45Ile (PDB or not)** | **SQSTM1** | 33.22 | 18.93 | 18.76 | 19.65 | 19.12 | 6.00 | 3 989 348 | 4 475 842 | **4 232 595** | 344 003 | 3 123 345 | 4 477 393 | 30 559 614 248 | 75 323 586 |
| 12 | **Carriers p.Pro392Leu** | **SQSTM1** | 34.83 | 18.89 | 18.88 | 20.03 | 19.30 | 6.00 | 4 343 802 | 4 373 169 | **4 358 485** | 20 766 | 2 418 401 | 3 953 130 | 30 559 614 248 | 66 354 811 |
| 13 | **Carriers p.Pro392Leu** | **SQSTM1** | 35.62 | 18.83 | 18.72 | 19.28 | 19.62 | 6.00 | 4 301 651 | 4 633 946 | **4 467 798** | 234 968 | 4 006 711 | 3 187 060 | 30 559 614 248 | 73 076 106 |
| 14 | **Carriers p.Pro392Leu** | **SQSTM1** | 33.41 | 19.81 | 19.55 | 21.21 | 19.59 | 6.00 | 2 162 188 | 2 578 805 | **2 370 497** | 294 593 | 1 092 847 | 3 252 076 | 30 559 614 248 | 47 711 459 |
| 15 | **Carriers p.Pro392Leu** | **SQSTM1** | 35.12 | 19.52 | 19.26 | 20.21 | 19.09 | 6.00 | 2 634 395 | 3 141 958 | **2 888 177** | 358 901 | 2 142 426 | 4 568 731 | 30 559 614 248 | 66 877 977 |
| 16 | **Carriers p.Pro392Leu** | **SQSTM1** | 45.00 | 20.92 | 21.00 | 20.58 | 21.11 | 6.00 | 1 317 104 | 1 247 801 | **1 282 452** | 49 005 | 2 067 686 | 1 447 241 | 37 835 076 995 | 48 377 122 |
| 17 | **Carriers p.Pro392Leu** | **SQSTM1** | 31.22 | 18.66 | 18.43 | 18.62 | 20.02 | 6.00 | 4 883 491 | 5 704 747 | **5 294 119** | 580 715 | 6 247 937 | 2 442 944 | 30 559 614 248 | 77 553 130 |
| 18 | **Carriers p.Pro392Leu** | **SQSTM1** | 35.60 | 19.28 | 19.28 | 19.78 | 19.26 | 6.00 | 3 212 171 | 3 212 171 | **3 212 171** | 0 | 2 861 639 | 4 074 710 | 30 559 614 248 | 70 895 687 |
| 19 | **Carriers p.Pro392Leu** | **SQSTM1** | 35.12 | 18.92 | 18.93 | 19.62 | 18.98 | 6.00 | 3 990 722 | 3 963 788 | **3 977 255** | 19 045 | 3 197 805 | 4 903 339 | 30 559 614 248 | 78 252 330 |
| 20 | **Healthy controls** | **SQSTM1** | 35.33 | 18.61 | 18.53 | 18.90 | 19.55 | 6.74 | 5 071 005 | 5 352 628 | **5 211 816** | 199 138 | 5 192 080 | 3 340 831 | 18 569 984 986 | 68 549 216 |
| 21 | **Healthy controls** | **SQSTM1** | 35.19 | 19.17 | 19.13 | 20.31 | 19.27 | 7.04 | 3 514 086 | 3 610 260 | **3 562 173** | 68 005 | 2 009 708 | 4 047 373 | 15 225 423 197 | 49 845 414 |
| 22 | **Healthy controls** | **SQSTM1** | 33.94 | 21.72 | 21.60 | 21.88 | 22.26 | 7.76 | 915 137 | 992 593 | **953 865** | 54 770 | 1 063 552 | 820 740 | 14 230 753 516 | 23 159 584 |
| 23 | **Healthy controls** | **SQSTM1** | 50.00 | 19.51 | 19.58 | 20.78 | 19.53 | 6.00 | 2 860 000 | 2 728 237 | **2 794 118** | 93 170 | 1 464 638 | 3 397 529 | 30 559 614 248 | 53 376 131 |
| 24 | **Healthy controls** | **SQSTM1** | 35.26 | 18.78 | 18.65 | 18.95 | 19.14 | 6.54 | 4 443 176 | 4 851 648 | **4 647 412** | 288 834 | 5 003 367 | 4 417 517 | 21 246 178 554 | 77 727 342 |
| 25 | **Healthy controls** | **SQSTM1** | 33.83 | 19.00 | 18.98 | 19.09 | 19.59 | 6.19 | 4 460 502 | 4 520 539 | **4 490 520** | 42 452 | 4 568 731 | 3 263 040 | 26 890 696 877 | 73 734 943 |
| 26 | **Healthy controls** | **SQSTM1** | 31.95 | 18.93 | 18.96 | 19.11 | 19.78 | 6.00 | 4 105 004 | 4 022 671 | **4 063 838** | 58 218 | 4 492 488 | 2 871 287 | 30 559 614 248 | 73 322 478 |
| 27 | **Healthy controls** | **SQSTM1** | 35.80 | 18.63 | 18.56 | 18.51 | 19.51 | 6.00 | 5 036 742 | 5 280 532 | **5 158 637** | 172 385 | 6 750 819 | 3 432 009 | 30 559 614 248 | 89 128 701 |
| 28 | **PDB non-mutated** | **SQSTM1** | 34.63 | 17.99 | 18.02 | 18.28 | 19.43 | 6.58 | 7 576 022 | 7 423 799 | **7 499 911** | 107 638 | 7 881 269 | 3 634 108 | 20 681 735 143 | 83 983 438 |
| 29 | **PDB non-mutated** | **SQSTM1** | 45.00 | 20.25 | 20.23 | 21.81 | 21.55 | 6.68 | 2 268 621 | 2 299 465 | **2 284 043** | 21 810 | 984 301 | 1 168 628 | 25 993 632 082 | 31 037 769 |
| 30 | **PDB non-mutated** | **SQSTM1** | 36.54 | 22.50 | 22.46 | 20.86 | 23.26 | 10.19 | 370 102 | 380 233 | **375 168** | 7 164 | 1 383 187 | 275 874 | 1 826 746 572 | 8 866 593 |
| 31 | **PDB non-mutated** | **SQSTM1** | 36.53 | 21.74 | 21.69 | 19.19 | 22.22 | 9.89 | 590 282 | 610 613 | **600 448** | 14 376 | 4 256 957 | 553 719 | 2 228 027 161 | 17 382 136 |
| 32 | **PDB non-mutated** | **SQSTM1** | 34.28 | 18.43 | 18.34 | 18.37 | 19.08 | 7.88 | 5 819 228 | 6 183 560 | **6 001 394** | 257 622 | 7 393 043 | 4 584 134 | 8 649 647 728 | 66 429 298 |
| 1 | **Carriers p.Val45Ile and p.Pro392Leu (PDB or not)** | **DOCK6** | 39.34 | 31.53 | 31.59 | 22.50 | 21.87 | 6.00 | 4 281 | 4 109 | **4 195** | 121 | 902 335 | 1 383 591 | 60 128 870 627 | 42 184 503 |
| 2 | **Healthy controls** | **DOCK6** | 45.00 | 28.67 | 28.67 | 21.57 | 19.03 | 6.00 | 15 509 | 15 509 | **15 509** | 0 | 857 659 | 4 741 051 | 30 559 614 248 | 49 901 368 |
| 3 | **Carriers p.Val45Ile (PDB or not)** | **DOCK6** | 45.00 | 30.63 | 30.99 | 21.29 | 22.41 | 6.00 | 6 662 | 5 214 | **5 938** | 1 024 | 1 724 905 | 811 591 | 50 902 747 621 | 41 458 593 |
| 4 | **Carriers p.Val45Ile and p.Pro392Leu (PDB or not)** | **DOCK6** | 34.06 | 27.44 | 27.46 | 21.17 | 20.08 | 6.00 | 35 347 | 34 869 | **35 108** | 338 | 1 126 458 | 2 346 241 | 30 559 614 248 | 43 226 018 |
| 5 | **Carriers p.Val45Ile and p.Pro392Leu (PDB or not)** | **DOCK6** | 34.66 | 27.87 | 27.64 | 19.08 | 19.12 | 6.00 | 26 727 | 31 252 | **28 989** | 3 199 | 4 584 134 | 4 477 393 | 30 559 614 248 | 85 600 598 |
| 6 | **Carriers p.Val45Ile and p.Pro392Leu (PDB or not)** | **DOCK6** | 36.77 | 27.85 | 27.88 | 18.73 | 19.57 | 6.00 | 27 817 | 27 256 | **27 537** | 397 | 5 821 572 | 3 296 155 | 30 559 614 248 | 83 701 245 |
| 7 | **Carriers p.Val45Ile (PDB or not)** | **DOCK6** | 34.95 | 28.39 | 28.07 | 19.08 | 19.69 | 6.00 | 18 163 | 22 586 | **20 374** | 3 128 | 4 584 134 | 3 050 618 | 30 559 614 248 | 75 323 586 |
| 8 | **Carriers p.Val45Ile (PDB or not)** | **DOCK6** | 33.55 | 27.71 | 27.51 | 18.49 | 19.63 | 6.00 | 28 671 | 32 857 | **30 764** | 2 960 | 6 842 321 | 3 176 351 | 30 559 614 248 | 87 248 897 |
| 9 | **Carriers p.Val45Ile (PDB or not)** | **DOCK6** | 33.16 | 27.61 | 27.51 | 18.70 | 19.20 | 6.00 | 30 357 | 32 499 | **31 428** | 1 514 | 5 940 331 | 4 228 397 | 30 559 614 248 | 91 561 191 |
| 10 | **Carriers p.Val45Ile (PDB or not)** | **DOCK6** | 34.37 | 27.20 | 27.44 | 19.50 | 19.37 | 6.00 | 42 211 | 35 856 | **39 033** | 4 494 | 3 466 838 | 3 783 891 | 30 559 614 248 | 73 734 943 |
| 11 | **Carriers p.Val45Ile (PDB or not)** | **DOCK6** | 33.22 | 28.49 | 28.58 | 19.65 | 19.12 | 6.00 | 16 510 | 15 527 | **16 019** | 695 | 3 123 345 | 4 477 393 | 30 559 614 248 | 75 323 586 |
| 12 | **Carriers p.Pro392Leu** | **DOCK6** | 34.83 | 28.80 | 28.67 | 20.03 | 19.30 | 6.00 | 14 610 | 15 958 | **15 284** | 953 | 2 418 401 | 3 953 130 | 30 559 614 248 | 66 354 811 |
| 13 | **Carriers p.Pro392Leu** | **DOCK6** | 35.62 | 28.19 | 28.20 | 19.28 | 19.62 | 6.00 | 20 495 | 20 356 | **20 425** | 98 | 4 006 711 | 3 187 060 | 30 559 614 248 | 73 076 106 |
| 14 | **Carriers p.Pro392Leu** | **DOCK6** | 33.41 | 27.41 | 27.23 | 21.21 | 19.59 | 6.00 | 33 686 | 38 092 | **35 889** | 3 116 | 1 092 847 | 3 252 076 | 30 559 614 248 | 47 711 459 |
| 15 | **Carriers p.Pro392Leu** | **DOCK6** | 35.12 | 28.51 | 28.74 | 20.21 | 19.09 | 6.00 | 15 916 | 13 603 | **14 759** | 1 636 | 2 142 426 | 4 568 731 | 30 559 614 248 | 66 877 977 |
| 16 | **Carriers p.Pro392Leu** | **DOCK6** | 45.00 | 30.53 | 30.22 | 20.58 | 21.11 | 6.00 | 5 276 | 6 516 | **5 896** | 877 | 2 067 686 | 1 447 241 | 37 835 076 995 | 48 377 122 |
| 17 | **Carriers p.Pro392Leu** | **DOCK6** | 31.22 | 28.08 | 28.00 | 18.62 | 20.02 | 6.00 | 22 492 | 23 751 | **23 122** | 891 | 6 247 937 | 2 442 944 | 30 559 614 248 | 77 553 130 |
| 18 | **Carriers p.Pro392Leu** | **DOCK6** | 35.60 | 28.56 | 28.63 | 19.78 | 19.26 | 6.00 | 16 223 | 15 467 | **15 845** | 534 | 2 861 639 | 4 074 710 | 30 559 614 248 | 70 895 687 |
| 19 | **Carriers p.Pro392Leu** | **DOCK6** | 35.12 | 28.32 | 28.33 | 19.62 | 18.98 | 6.00 | 18 361 | 18 236 | **18 299** | 88 | 3 197 805 | 4 903 339 | 30 559 614 248 | 78 252 330 |
| 20 | **Healthy controls** | **DOCK6** | 35.33 | 28.24 | 28.20 | 18.90 | 19.55 | 6.74 | 20 290 | 20 850 | **20 570** | 396 | 5 192 080 | 3 340 831 | 18 569 984 986 | 68 549 216 |
| 21 | **Healthy controls** | **DOCK6** | 35.19 | 28.15 | 27.85 | 20.31 | 19.27 | 7.04 | 21 945 | 26 913 | **24 429** | 3 513 | 2 009 708 | 4 047 373 | 15 225 423 197 | 49 845 414 |
| 22 | **Healthy controls** | **DOCK6** | 33.94 | 30.69 | 30.90 | 21.88 | 22.26 | 7.76 | 5 572 | 4 828 | **5 200** | 526 | 1 063 552 | 820 740 | 14 230 753 516 | 23 159 584 |
| 23 | **Healthy controls** | **DOCK6** | 50.00 | 28.54 | 28.47 | 20.78 | 19.53 | 6.00 | 17 426 | 18 274 | **17 850** | 600 | 1 464 638 | 3 397 529 | 30 559 614 248 | 53 376 131 |
| 24 | **Healthy controls** | **DOCK6** | 35.26 | 27.83 | 27.81 | 18.95 | 19.14 | 6.54 | 26 138 | 26 497 | **26 318** | 254 | 5 003 367 | 4 417 517 | 21 246 178 554 | 77 727 342 |
| 25 | **Healthy controls** | **DOCK6** | 33.83 | 28.13 | 28.05 | 19.09 | 19.59 | 6.19 | 26 756 | 28 237 | **27 496** | 1 048 | 4 568 731 | 3 263 040 | 26 890 696 877 | 73 734 943 |
| 26 | **Healthy controls** | **DOCK6** | 31.95 | 28.22 | 28.27 | 19.11 | 19.78 | 6.00 | 20 719 | 20 026 | **20 373** | 490 | 4 492 488 | 2 871 287 | 30 559 614 248 | 73 322 478 |
| 27 | **Healthy controls** | **DOCK6** | 35.80 | 28.13 | 28.18 | 18.51 | 19.51 | 6.00 | 22 093 | 21 354 | **21 724** | 523 | 6 750 819 | 3 432 009 | 30 559 614 248 | 89 128 701 |
| 28 | **PDB non-mutated** | **DOCK6** | 34.63 | 28.43 | 28.48 | 18.28 | 19.43 | 6.58 | 17 340 | 16 759 | **17 050** | 411 | 7 881 269 | 3 634 108 | 20 681 735 143 | 83 983 438 |
| 29 | **PDB non-mutated** | **DOCK6** | 45.00 | 31.16 | 31.71 | 21.81 | 21.55 | 6.68 | 3 781 | 2 601 | **3 191** | 835 | 984 301 | 1 168 628 | 25 993 632 082 | 31 037 769 |
| 30 | **PDB non-mutated** | **DOCK6** | 36.54 | 29.64 | 29.43 | 20.86 | 23.26 | 10.19 | 7 933 | 9 152 | **8 543** | 862 | 1 383 187 | 275 874 | 1 826 746 572 | 8 866 593 |
| 31 | **PDB non-mutated** | **DOCK6** | 36.53 | 29.07 | 29.10 | 19.19 | 22.22 | 9.89 | 10 985 | 10 762 | **10 873** | 157 | 4 256 957 | 553 719 | 2 228 027 161 | 17 382 136 |
| 32 | **PDB non-mutated** | **DOCK6** | 34.28 | 27.72 | 27.68 | 18.37 | 19.08 | 7.88 | 29 619 | 30 436 | **30 028** | 577 | 7 393 043 | 4 584 134 | 8 649 647 728 | 66 429 298 |
| 1 | **Carriers p.Val45Ile and p.Pro392Leu (PDB or not)** | **DOCK5** | 39.34 | 25.57 | 25.40 | 22.50 | 21.87 | 6.00 | 80 648 | 90 555 | **85 601** | 7 006 | 902 335 | 1 383 591 | 60 128 870 627 | 42 184 503 |
| 2 | **Healthy controls** | **DOCK5** | 45.00 | 24.03 | 23.95 | 21.57 | 19.03 | 6.00 | 118 839 | 125 493 | **122 166** | 4 705 | 857 659 | 4 741 051 | 30 559 614 248 | 49 901 368 |
| 3 | **Carriers p.Val45Ile (PDB or not)** | **DOCK5** | 45.00 | 25.24 | 25.15 | 21.29 | 22.41 | 6.00 | 85 237 | 90 630 | **87 933** | 3 814 | 1 724 905 | 811 591 | 50 902 747 621 | 41 458 593 |
| 4 | **Carriers p.Val45Ile and p.Pro392Leu (PDB or not)** | **DOCK5** | 34.06 | 22.94 | 22.94 | 21.17 | 20.08 | 6.00 | 247 027 | 247 027 | **247 027** | 0 | 1 126 458 | 2 346 241 | 30 559 614 248 | 43 226 018 |
| 5 | **Carriers p.Val45Ile and p.Pro392Leu (PDB or not)** | **DOCK5** | 34.66 | 22.92 | 23.39 | 19.08 | 19.12 | 6.00 | 253 125 | 183 797 | **218 461** | 49 022 | 4 584 134 | 4 477 393 | 30 559 614 248 | 85 600 598 |
| 6 | **Carriers p.Val45Ile and p.Pro392Leu (PDB or not)** | **DOCK5** | 36.77 | 23.64 | 23.57 | 18.73 | 19.57 | 6.00 | 158 541 | 166 271 | **162 406** | 5 465 | 5 821 572 | 3 296 155 | 30 559 614 248 | 83 701 245 |
| 7 | **Carriers p.Val45Ile (PDB or not)** | **DOCK5** | 34.95 | 23.60 | 23.76 | 19.08 | 19.69 | 6.00 | 155 023 | 138 994 | **147 009** | 11 334 | 4 584 134 | 3 050 618 | 30 559 614 248 | 75 323 586 |
| 8 | **Carriers p.Val45Ile (PDB or not)** | **DOCK5** | 33.55 | 22.71 | 22.59 | 18.49 | 19.63 | 6.00 | 282 934 | 307 076 | **295 005** | 17 071 | 6 842 321 | 3 176 351 | 30 559 614 248 | 87 248 897 |
| 9 | **Carriers p.Val45Ile (PDB or not)** | **DOCK5** | 33.16 | 22.57 | 22.37 | 18.70 | 19.20 | 6.00 | 308 508 | 353 647 | **331 077** | 31 918 | 5 940 331 | 4 228 397 | 30 559 614 248 | 91 561 191 |
| 10 | **Carriers p.Val45Ile (PDB or not)** | **DOCK5** | 34.37 | 23.46 | 23.23 | 19.50 | 19.37 | 6.00 | 175 457 | 205 204 | **190 331** | 21 034 | 3 466 838 | 3 783 891 | 30 559 614 248 | 73 734 943 |
| 11 | **Carriers p.Val45Ile (PDB or not)** | **DOCK5** | 33.22 | 23.54 | 23.24 | 19.65 | 19.12 | 6.00 | 157 893 | 193 803 | **175 848** | 25 392 | 3 123 345 | 4 477 393 | 30 559 614 248 | 75 323 586 |
| 12 | **Carriers p.Pro392Leu** | **DOCK5** | 34.83 | 23.30 | 23.16 | 20.03 | 19.30 | 6.00 | 199 959 | 219 930 | **209 945** | 14 122 | 2 418 401 | 3 953 130 | 30 559 614 248 | 66 354 811 |
| 13 | **Carriers p.Pro392Leu** | **DOCK5** | 35.62 | 23.13 | 23.44 | 19.28 | 19.62 | 6.00 | 210 926 | 170 695 | **190 811** | 28 447 | 4 006 711 | 3 187 060 | 30 559 614 248 | 73 076 106 |
| 14 | **Carriers p.Pro392Leu** | **DOCK5** | 33.41 | 23.16 | 23.56 | 21.21 | 19.59 | 6.00 | 200 655 | 152 629 | **176 642** | 33 959 | 1 092 847 | 3 252 076 | 30 559 614 248 | 47 711 459 |
| 15 | **Carriers p.Pro392Leu** | **DOCK5** | 35.12 | 22.91 | 22.83 | 20.21 | 19.09 | 6.00 | 238 352 | 251 756 | **245 054** | 9 478 | 2 142 426 | 4 568 731 | 30 559 614 248 | 66 877 977 |
| 16 | **Carriers p.Pro392Leu** | **DOCK5** | 45.00 | 24.18 | 24.27 | 20.58 | 21.11 | 6.00 | 130 023 | 122 283 | **126 153** | 5 472 | 2 067 686 | 1 447 241 | 37 835 076 995 | 48 377 122 |
| 17 | **Carriers p.Pro392Leu** | **DOCK5** | 31.22 | 22.51 | 22.56 | 18.62 | 20.02 | 6.00 | 326 752 | 315 797 | **321 275** | 7 746 | 6 247 937 | 2 442 944 | 30 559 614 248 | 77 553 130 |
| 18 | **Carriers p.Pro392Leu** | **DOCK5** | 35.60 | 24.01 | 23.92 | 19.78 | 19.26 | 6.00 | 117 482 | 124 919 | **121 201** | 5 259 | 2 861 639 | 4 074 710 | 30 559 614 248 | 70 895 687 |
| 19 | **Carriers p.Pro392Leu** | **DOCK5** | 35.12 | 23.46 | 23.33 | 19.62 | 18.98 | 6.00 | 165 426 | 180 796 | **173 111** | 10 868 | 3 197 805 | 4 903 339 | 30 559 614 248 | 78 252 330 |
| 20 | **Healthy controls** | **DOCK5** | 35.33 | 23.78 | 23.77 | 18.90 | 19.55 | 6.74 | 138 109 | 139 054 | **138 582** | 668 | 5 192 080 | 3 340 831 | 18 569 984 986 | 68 549 216 |
| 21 | **Healthy controls** | **DOCK5** | 35.19 | 23.20 | 23.14 | 20.31 | 19.27 | 7.04 | 208 022 | 216 700 | **212 361** | 6 137 | 2 009 708 | 4 047 373 | 15 225 423 197 | 49 845 414 |
| 22 | **Healthy controls** | **DOCK5** | 33.94 | 24.77 | 24.77 | 21.88 | 22.26 | 7.76 | 103 257 | 103 257 | **103 257** | 0 | 1 063 552 | 820 740 | 14 230 753 516 | 23 159 584 |
| 23 | **Healthy controls** | **DOCK5** | 50.00 | 23.72 | 23.72 | 20.78 | 19.53 | 6.00 | 150 251 | 150 251 | **150 251** | 0 | 1 464 638 | 3 397 529 | 30 559 614 248 | 53 376 131 |
| 24 | **Healthy controls** | **DOCK5** | 35.26 | 23.13 | 23.47 | 18.95 | 19.14 | 6.54 | 210 545 | 166 929 | **188 737** | 30 841 | 5 003 367 | 4 417 517 | 21 246 178 554 | 77 727 342 |
| 25 | **Healthy controls** | **DOCK5** | 33.83 | 23.59 | 23.34 | 19.09 | 19.59 | 6.19 | 186 234 | 220 448 | **203 341** | 24 193 | 4 568 731 | 3 263 040 | 26 890 696 877 | 73 734 943 |
| 26 | **Healthy controls** | **DOCK5** | 31.95 | 23.67 | 23.27 | 19.11 | 19.78 | 6.00 | 149 782 | 196 724 | **173 253** | 33 193 | 4 492 488 | 2 871 287 | 30 559 614 248 | 73 322 478 |
| 27 | **Healthy controls** | **DOCK5** | 35.80 | 23.28 | 23.18 | 18.51 | 19.51 | 6.00 | 195 868 | 209 681 | **202 775** | 9 767 | 6 750 819 | 3 432 009 | 30 559 614 248 | 89 128 701 |
| 28 | **PDB non-mutated** | **DOCK5** | 34.63 | 23.64 | 23.64 | 18.28 | 19.43 | 6.58 | 148 471 | 148 471 | **148 471** | 0 | 7 881 269 | 3 634 108 | 20 681 735 143 | 83 983 438 |
| 29 | **PDB non-mutated** | **DOCK5** | 45.00 | 25.04 | 25.05 | 21.81 | 21.55 | 6.68 | 79 401 | 78 862 | **79 131** | 381 | 984 301 | 1 168 628 | 25 993 632 082 | 31 037 769 |
| 30 | **PDB non-mutated** | **DOCK5** | 36.54 | 24.99 | 25.10 | 20.86 | 23.26 | 10.19 | 61 249 | 56 826 | **59 037** | 3 127 | 1 383 187 | 275 874 | 1 826 746 572 | 8 866 593 |
| 31 | **PDB non-mutated** | **DOCK5** | 36.53 | 25.07 | 25.09 | 19.19 | 22.22 | 9.89 | 54 958 | 54 212 | **54 585** | 528 | 4 256 957 | 553 719 | 2 228 027 161 | 17 382 136 |
| 32 | **PDB non-mutated** | **DOCK5** | 34.28 | 22.99 | 22.89 | 18.37 | 19.08 | 7.88 | 241 494 | 258 511 | **250 002** | 12 033 | 7 393 043 | 4 584 134 | 8 649 647 728 | 66 429 298 |
| 1 | **Carriers p.Val45Ile and p.Pro392Leu (PDB or not)** | **DOCK2** | 39.34 | 25.41 | 25.34 | 22.50 | 21.87 | 6.00 | 182 116 | 189 893 | **186 005** | 5 499 | 902 335 | 1 383 591 | 60 128 870 627 | 42 184 503 |
| 2 | **Healthy controls** | **DOCK2** | 45.00 | 23.42 | 23.26 | 21.57 | 19.03 | 6.00 | 307 757 | 338 596 | **323 177** | 21 806 | 857 659 | 4 741 051 | 30 559 614 248 | 49 901 368 |
| 3 | **Carriers p.Val45Ile (PDB or not)** | **DOCK2** | 45.00 | 26.59 | 26.55 | 21.29 | 22.41 | 6.00 | 75 973 | 77 811 | **76 892** | 1 299 | 1 724 905 | 811 591 | 50 902 747 621 | 41 458 593 |
| 4 | **Carriers p.Val45Ile and p.Pro392Leu (PDB or not)** | **DOCK2** | 34.06 | 23.71 | 23.66 | 21.17 | 20.08 | 6.00 | 256 387 | 264 159 | **260 273** | 5 495 | 1 126 458 | 2 346 241 | 30 559 614 248 | 43 226 018 |
| 5 | **Carriers p.Val45Ile and p.Pro392Leu (PDB or not)** | **DOCK2** | 34.66 | 23.16 | 23.17 | 19.08 | 19.12 | 6.00 | 359 494 | 357 355 | **358 425** | 1 513 | 4 584 134 | 4 477 393 | 30 559 614 248 | 85 600 598 |
| 6 | **Carriers p.Val45Ile and p.Pro392Leu (PDB or not)** | **DOCK2** | 36.77 | 23.50 | 23.71 | 18.73 | 19.57 | 6.00 | 299 260 | 264 054 | **281 657** | 24 895 | 5 821 572 | 3 296 155 | 30 559 614 248 | 83 701 245 |
| 7 | **Carriers p.Val45Ile (PDB or not)** | **DOCK2** | 34.95 | 23.97 | 23.93 | 19.08 | 19.69 | 6.00 | 216 372 | 221 608 | **218 990** | 3 703 | 4 584 134 | 3 050 618 | 30 559 614 248 | 75 323 586 |
| 8 | **Carriers p.Val45Ile (PDB or not)** | **DOCK2** | 33.55 | 23.54 | 23.49 | 18.49 | 19.63 | 6.00 | 278 419 | 286 870 | **282 645** | 5 976 | 6 842 321 | 3 176 351 | 30 559 614 248 | 87 248 897 |
| 9 | **Carriers p.Val45Ile (PDB or not)** | **DOCK2** | 33.16 | 22.50 | 22.57 | 18.70 | 19.20 | 6.00 | 514 527 | 493 419 | **503 973** | 14 925 | 5 940 331 | 4 228 397 | 30 559 614 248 | 91 561 191 |
| 10 | **Carriers p.Val45Ile (PDB or not)** | **DOCK2** | 34.37 | 24.08 | 23.97 | 19.50 | 19.37 | 6.00 | 207 829 | 221 931 | **214 880** | 9 971 | 3 466 838 | 3 783 891 | 30 559 614 248 | 73 734 943 |
| 11 | **Carriers p.Val45Ile (PDB or not)** | **DOCK2** | 33.22 | 22.56 | 22.81 | 19.65 | 19.12 | 6.00 | 493 240 | 424 675 | **458 958** | 48 483 | 3 123 345 | 4 477 393 | 30 559 614 248 | 75 323 586 |
| 12 | **Carriers p.Pro392Leu** | **DOCK2** | 34.83 | 23.49 | 23.49 | 20.03 | 19.30 | 6.00 | 301 287 | 301 287 | **301 287** | 0 | 2 418 401 | 3 953 130 | 30 559 614 248 | 66 354 811 |
| 13 | **Carriers p.Pro392Leu** | **DOCK2** | 35.62 | 24.12 | 24.45 | 19.28 | 19.62 | 6.00 | 195 537 | 160 501 | **178 019** | 24 774 | 4 006 711 | 3 187 060 | 30 559 614 248 | 73 076 106 |
| 14 | **Carriers p.Pro392Leu** | **DOCK2** | 33.41 | 24.71 | 24.67 | 21.21 | 19.59 | 6.00 | 133 647 | 136 891 | **135 269** | 2 293 | 1 092 847 | 3 252 076 | 30 559 614 248 | 47 711 459 |
| 15 | **Carriers p.Pro392Leu** | **DOCK2** | 35.12 | 23.16 | 23.19 | 20.21 | 19.09 | 6.00 | 338 792 | 332 754 | **335 773** | 4 269 | 2 142 426 | 4 568 731 | 30 559 614 248 | 66 877 977 |
| 16 | **Carriers p.Pro392Leu** | **DOCK2** | 45.00 | 25.86 | 25.86 | 20.58 | 21.11 | 6.00 | 87 046 | 87 046 | **87 046** | 0 | 2 067 686 | 1 447 241 | 37 835 076 995 | 48 377 122 |
| 17 | **Carriers p.Pro392Leu** | **DOCK2** | 31.22 | 23.91 | 23.89 | 18.62 | 20.02 | 6.00 | 224 718 | 227 420 | **226 069** | 1 911 | 6 247 937 | 2 442 944 | 30 559 614 248 | 77 553 130 |
| 18 | **Carriers p.Pro392Leu** | **DOCK2** | 35.60 | 22.95 | 22.98 | 19.78 | 19.26 | 6.00 | 398 935 | 391 844 | **395 390** | 5 014 | 2 861 639 | 4 074 710 | 30 559 614 248 | 70 895 687 |
| 19 | **Carriers p.Pro392Leu** | **DOCK2** | 35.12 | 22.91 | 23.12 | 19.62 | 18.98 | 6.00 | 397 253 | 350 299 | **373 776** | 33 202 | 3 197 805 | 4 903 339 | 30 559 614 248 | 78 252 330 |
| 20 | **Healthy controls** | **DOCK2** | 35.33 | 23.83 | 24.00 | 18.90 | 19.55 | 6.74 | 236 765 | 213 894 | **225 329** | 16 172 | 5 192 080 | 3 340 831 | 18 569 984 986 | 68 549 216 |
| 21 | **Healthy controls** | **DOCK2** | 35.19 | 22.34 | 22.51 | 20.31 | 19.27 | 7.04 | 583 708 | 527 371 | **555 540** | 39 837 | 2 009 708 | 4 047 373 | 15 225 423 197 | 49 845 414 |
| 22 | **Healthy controls** | **DOCK2** | 33.94 | 26.42 | 26.50 | 21.88 | 22.26 | 7.76 | 74 133 | 70 665 | **72 399** | 2 452 | 1 063 552 | 820 740 | 14 230 753 516 | 23 159 584 |
| 23 | **Healthy controls** | **DOCK2** | 50.00 | 24.22 | 24.16 | 20.78 | 19.53 | 6.00 | 194 963 | 202 060 | **198 511** | 5 019 | 1 464 638 | 3 397 529 | 30 559 614 248 | 53 376 131 |
| 24 | **Healthy controls** | **DOCK2** | 35.26 | 22.77 | 22.74 | 18.95 | 19.14 | 6.54 | 437 875 | 445 807 | **441 841** | 5 609 | 5 003 367 | 4 417 517 | 21 246 178 554 | 77 727 342 |
| 25 | **Healthy controls** | **DOCK2** | 33.83 | 23.54 | 23.50 | 19.09 | 19.59 | 6.19 | 326 535 | 334 350 | **330 443** | 5 526 | 4 568 731 | 3 263 040 | 26 890 696 877 | 73 734 943 |
| 26 | **Healthy controls** | **DOCK2** | 31.95 | 23.76 | 23.76 | 19.11 | 19.78 | 6.00 | 248 216 | 248 216 | **248 216** | 0 | 4 492 488 | 2 871 287 | 30 559 614 248 | 73 322 478 |
| 27 | **Healthy controls** | **DOCK2** | 35.80 | 23.70 | 23.81 | 18.51 | 19.51 | 6.00 | 257 838 | 241 442 | **249 640** | 11 593 | 6 750 819 | 3 432 009 | 30 559 614 248 | 89 128 701 |
| 28 | **PDB non-mutated** | **DOCK2** | 34.63 | 23.62 | 23.64 | 18.28 | 19.43 | 6.58 | 263 044 | 259 914 | **261 479** | 2 213 | 7 881 269 | 3 634 108 | 20 681 735 143 | 83 983 438 |
| 29 | **PDB non-mutated** | **DOCK2** | 45.00 | 25.58 | 25.48 | 21.81 | 21.55 | 6.68 | 112 825 | 119 769 | **116 297** | 4 910 | 984 301 | 1 168 628 | 25 993 632 082 | 31 037 769 |
| 30 | **PDB non-mutated** | **DOCK2** | 36.54 | 26.07 | 26.11 | 20.86 | 23.26 | 10.19 | 62 763 | 61 281 | **62 022** | 1 048 | 1 383 187 | 275 874 | 1 826 746 572 | 8 866 593 |
| 31 | **PDB non-mutated** | **DOCK2** | 36.53 | 25.94 | 25.85 | 19.19 | 22.22 | 9.89 | 64 595 | 68 174 | **66 384** | 2 530 | 4 256 957 | 553 719 | 2 228 027 161 | 17 382 136 |
| 32 | **PDB non-mutated** | **DOCK2** | 34.28 | 23.04 | 23.00 | 18.37 | 19.08 | 7.88 | 386 399 | 395 734 | **391 066** | 6 601 | 7 393 043 | 4 584 134 | 8 649 647 728 | 66 429 298 |
| 1 | **Carriers p.Val45Ile and p.Pro392Leu (PDB or not)** | **RAC1** | 39.34 | 22.89 | 23.01 | 22.50 | 21.87 | 6.00 | 2 439 437 | 2 269 493 | **2 354 465** | 120 169 | 902 335 | 1 383 591 | 60 128 870 627 | 42 184 503 |
| 2 | **Healthy controls** | **RAC1** | 45.00 | 21.65 | 21.66 | 21.57 | 19.03 | 6.00 | 2 645 725 | 2 629 866 | **2 637 796** | 11 214 | 857 659 | 4 741 051 | 30 559 614 248 | 49 901 368 |
| 3 | **Carriers p.Val45Ile (PDB or not)** | **RAC1** | 45.00 | 23.66 | 23.45 | 21.29 | 22.41 | 6.00 | 1 296 089 | 1 470 706 | **1 383 398** | 123 473 | 1 724 905 | 811 591 | 50 902 747 621 | 41 458 593 |
| 4 | **Carriers p.Val45Ile and p.Pro392Leu (PDB or not)** | **RAC1** | 34.06 | 22.51 | 22.16 | 21.17 | 20.08 | 6.00 | 1 563 268 | 1 929 662 | **1 746 465** | 259 080 | 1 126 458 | 2 346 241 | 30 559 614 248 | 43 226 018 |
| 5 | **Carriers p.Val45Ile and p.Pro392Leu (PDB or not)** | **RAC1** | 34.66 | 20.72 | 21.10 | 19.08 | 19.12 | 6.00 | 4 628 625 | 3 683 274 | **4 155 950** | 668 464 | 4 584 134 | 4 477 393 | 30 559 614 248 | 85 600 598 |
| 6 | **Carriers p.Val45Ile and p.Pro392Leu (PDB or not)** | **RAC1** | 36.77 | 20.99 | 20.62 | 18.73 | 19.57 | 6.00 | 4 004 886 | 5 001 065 | **4 502 976** | 704 405 | 5 821 572 | 3 296 155 | 30 559 614 248 | 83 701 245 |
| 7 | **Carriers p.Val45Ile (PDB or not)** | **RAC1** | 34.95 | 20.79 | 20.75 | 19.08 | 19.69 | 6.00 | 4 344 791 | 4 450 723 | **4 397 757** | 74 905 | 4 584 134 | 3 050 618 | 30 559 614 248 | 75 323 586 |
| 8 | **Carriers p.Val45Ile (PDB or not)** | **RAC1** | 33.55 | 20.47 | 20.45 | 18.49 | 19.63 | 6.00 | 5 245 432 | 5 309 015 | **5 277 224** | 44 960 | 6 842 321 | 3 176 351 | 30 559 614 248 | 87 248 897 |
| 9 | **Carriers p.Val45Ile (PDB or not)** | **RAC1** | 33.16 | 20.97 | 21.04 | 18.70 | 19.20 | 6.00 | 3 852 649 | 3 693 467 | **3 773 058** | 112 558 | 5 940 331 | 4 228 397 | 30 559 614 248 | 91 561 191 |
| 10 | **Carriers p.Val45Ile (PDB or not)** | **RAC1** | 34.37 | 20.76 | 21.10 | 19.50 | 19.37 | 6.00 | 4 522 995 | 3 686 880 | **4 104 938** | 591 223 | 3 466 838 | 3 783 891 | 30 559 614 248 | 73 734 943 |
| 11 | **Carriers p.Val45Ile (PDB or not)** | **RAC1** | 33.22 | 20.64 | 20.58 | 19.65 | 19.12 | 6.00 | 4 673 139 | 4 845 330 | **4 759 235** | 121 757 | 3 123 345 | 4 477 393 | 30 559 614 248 | 75 323 586 |
| 12 | **Carriers p.Pro392Leu** | **RAC1** | 34.83 | 21.12 | 20.88 | 20.03 | 19.30 | 6.00 | 3 706 848 | 4 281 322 | **3 994 085** | 406 215 | 2 418 401 | 3 953 130 | 30 559 614 248 | 66 354 811 |
| 13 | **Carriers p.Pro392Leu** | **RAC1** | 35.62 | 21.02 | 20.99 | 19.28 | 19.62 | 6.00 | 3 744 572 | 3 812 894 | **3 778 733** | 48 311 | 4 006 711 | 3 187 060 | 30 559 614 248 | 73 076 106 |
| 14 | **Carriers p.Pro392Leu** | **RAC1** | 33.41 | 22.35 | 22.22 | 21.21 | 19.59 | 6.00 | 1 638 241 | 1 772 022 | **1 705 131** | 94 597 | 1 092 847 | 3 252 076 | 30 559 614 248 | 47 711 459 |
| 15 | **Carriers p.Pro392Leu** | **RAC1** | 35.12 | 20.93 | 20.86 | 20.21 | 19.09 | 6.00 | 3 865 198 | 4 032 062 | **3 948 630** | 117 990 | 2 142 426 | 4 568 731 | 30 559 614 248 | 66 877 977 |
| 16 | **Carriers p.Pro392Leu** | **RAC1** | 45.00 | 22.08 | 21.89 | 20.58 | 21.11 | 6.00 | 2 485 987 | 2 787 233 | **2 636 610** | 213 013 | 2 067 686 | 1 447 241 | 37 835 076 995 | 48 377 122 |
| 17 | **Carriers p.Pro392Leu** | **RAC1** | 31.22 | 20.89 | 20.97 | 18.62 | 20.02 | 6.00 | 4 097 977 | 3 905 252 | **4 001 615** | 136 278 | 6 247 937 | 2 442 944 | 30 559 614 248 | 77 553 130 |
| 18 | **Carriers p.Pro392Leu** | **RAC1** | 35.60 | 20.70 | 20.63 | 19.78 | 19.26 | 6.00 | 4 595 099 | 4 792 920 | **4 694 010** | 139 881 | 2 861 639 | 4 074 710 | 30 559 614 248 | 70 895 687 |
| 19 | **Carriers p.Pro392Leu** | **RAC1** | 35.12 | 20.86 | 20.74 | 19.62 | 18.98 | 6.00 | 4 066 752 | 4 372 126 | **4 219 439** | 215 932 | 3 197 805 | 4 903 339 | 30 559 614 248 | 78 252 330 |
| 20 | **Healthy controls** | **RAC1** | 35.33 | 21.15 | 21.17 | 18.90 | 19.55 | 6.74 | 3 517 932 | 3 475 834 | **3 496 883** | 29 768 | 5 192 080 | 3 340 831 | 18 569 984 986 | 68 549 216 |
| 21 | **Healthy controls** | **RAC1** | 35.19 | 20.85 | 20.80 | 20.31 | 19.27 | 7.04 | 4 261 765 | 4 391 867 | **4 326 816** | 91 995 | 2 009 708 | 4 047 373 | 15 225 423 197 | 49 845 414 |
| 22 | **Healthy controls** | **RAC1** | 33.94 | 23.65 | 23.65 | 21.88 | 22.26 | 7.76 | 1 153 560 | 1 153 560 | **1 153 560** | 0 | 1 063 552 | 820 740 | 14 230 753 516 | 23 159 584 |
| 23 | **Healthy controls** | **RAC1** | 50.00 | 21.49 | 21.55 | 20.78 | 19.53 | 6.00 | 2 967 984 | 2 862 978 | **2 915 481** | 74 250 | 1 464 638 | 3 397 529 | 30 559 614 248 | 53 376 131 |
| 24 | **Healthy controls** | **RAC1** | 35.26 | 20.77 | 20.69 | 18.95 | 19.14 | 6.54 | 4 347 293 | 4 562 065 | **4 454 679** | 151 867 | 5 003 367 | 4 417 517 | 21 246 178 554 | 77 727 342 |
| 25 | **Healthy controls** | **RAC1** | 33.83 | 20.95 | 21.01 | 19.09 | 19.59 | 6.19 | 4 531 704 | 4 372 614 | **4 452 159** | 112 493 | 4 568 731 | 3 263 040 | 26 890 696 877 | 73 734 943 |
| 26 | **Healthy controls** | **RAC1** | 31.95 | 21.02 | 21.01 | 19.11 | 19.78 | 6.00 | 3 822 630 | 3 845 701 | **3 834 165** | 16 314 | 4 492 488 | 2 871 287 | 30 559 614 248 | 73 322 478 |
| 27 | **Healthy controls** | **RAC1** | 35.80 | 21.11 | 21.07 | 18.51 | 19.51 | 6.00 | 3 628 244 | 3 716 618 | **3 672 431** | 62 490 | 6 750 819 | 3 432 009 | 30 559 614 248 | 89 128 701 |
| 28 | **PDB non-mutated** | **RAC1** | 34.63 | 20.91 | 20.92 | 18.28 | 19.43 | 6.58 | 3 992 005 | 3 968 013 | **3 980 009** | 16 965 | 7 881 269 | 3 634 108 | 20 681 735 143 | 83 983 438 |
| 29 | **PDB non-mutated** | **RAC1** | 45.00 | 22.84 | 22.81 | 21.81 | 21.55 | 6.68 | 1 723 299 | 1 754 685 | **1 738 992** | 22 193 | 984 301 | 1 168 628 | 25 993 632 082 | 31 037 769 |
| 30 | **PDB non-mutated** | **RAC1** | 36.54 | 24.48 | 24.32 | 20.86 | 23.26 | 10.19 | 478 857 | 527 235 | **503 046** | 34 209 | 1 383 187 | 275 874 | 1 826 746 572 | 8 866 593 |
| 31 | **PDB non-mutated** | **RAC1** | 36.53 | 23.53 | 23.49 | 19.19 | 22.22 | 9.89 | 810 965 | 830 778 | **820 872** | 14 010 | 4 256 957 | 553 719 | 2 228 027 161 | 17 382 136 |
| 32 | **PDB non-mutated** | **RAC1** | 34.28 | 20.78 | 20.61 | 18.37 | 19.08 | 7.88 | 4 466 876 | 4 947 537 | **4 707 206** | 339 879 | 7 393 043 | 4 584 134 | 8 649 647 728 | 66 429 298 |

**Supplementary table 6:** Raw data of the protein expression analyses by Western Blot

| Categories | GEL 1 | DOCK6 | VINCULIN | RATIO | DOCK5 | VINCULIN | RATIO | DOCK2 | VINCULIN | RATIO | RAC1 | TUBULINE | RATIO | SQSTM1 | TUBULINE | RATIO |
| --- | --- | --- | --- | --- | --- | --- | --- | --- | --- | --- | --- | --- | --- | --- | --- | --- |
| 1 | PDB SQSTM1 +DOCK6+ | 53600 | 759000 | 0.07061924 | 54826 | 759000 | 0.07223452 | 131036 | 337676 | 0.38805245 | 7280 | 585486 | 0.01243411 | 730380 | 769227 | 0.94949865 |
|  |  | 215826 | 800064 | 0.26976092 | 159936 | 800064 | 0.19990401 | 283032 | 252525 | 1.12080784 | 153881 | 722432 | 0.21300413 | 1126540 | 1166980 | 0.96534645 |
|  |  | | | | | | | | | | | | | | | |
| 1 | carrier SQSTM1+ DOCK6+ | 56990 | 691380 | 0.08242934 | 7272 | 691380 | 0.01051809 | 86430 | 314358 | 0.27494131 | 91170 | 65533 | 1.39120748 | 686000 | 689214 | 0.99533672 |
|  |  | 15392 | 131328 | 0.11720273 | 7950 | 131328 | 0.06053545 | 57393 | 300498 | 0.19099295 | 17490 | 255341 | 0.06849664 | 664200 | 659430 | 1.00723352 |
|  |  | | | | | | | | | | | | | | | |
| 2 | PDB SQSTM1-DOCK6+ | 52605 | 604236 | 0.08706035 | 10787 | 604236 | 0.0178523 | 71980 | 331397 | 0.21720172 | 26226 | 609756 | 0.04301065 | 835883 | 695 385 | 1.20204347 |
|  |  |  |  |  |  |  |  |  |  |  |  |  |  |  |  |  |
|  | GEL 2 | DOCK6 | VINCULIN | RATIO | DOCK5 | VINCULIN | RATIO | DOCK2 | VINCULIN | RATIO | RAC1 | TUBULINE | RATIO | SQSTM1 | TUBULINE | RATIO |
| 2 | carrier SQSTM1-DOCK6+ | 50107 | 151226 | 0.33133853 | 8395 | 151226 | 0.05551294 | 78183 | 162060 | 0.48243243 | 42336 | 474075 | 0.08930233 | 689481 | 770695 | 0.89462239 |
|  |  | 65102 | 379500 | 0.17154677 | 31950 | 379500 | 0.08418972 | 110885 | 357192 | 0.31043528 | 78812 | 752544 | 0.10472743 | 760185 | 778320 | 0.97669981 |
|  |  | 57583 | 363970 | 0.15820809 | 40104 | 363970 | 0.11018491 | 115240 | 451872 | 0.25502797 | 7215 | 680330 | 0.01060515 | 870000 | 857108 | 1.01504128 |
|  |  | 37736 | 204097 | 0.18489248 | 27255 | 204097 | 0.13353944 | 69048 | 171801 | 0.40190686 | 10855 | 564416 | 0.01923227 | 663432 | 655098 | 1.01272176 |
|  |  | 138039 | 32204 | 4.28639299 | 0 | 32204 | 0 | 14272 | 347882 | 0.04102541 | 0 | 0 | 0 | 0 | 260537 | 0 |
|  |  |  |  |  |  |  |  |  |  |  |  |  |  |  |  |  |
|  | GEL 3 | DOCK6 | VINCULIN | RATIO | DOCK5 | VINCULIN | RATIO | DOCK2 | VINCULIN | RATIO | RAC1 | TUBULINE | RATIO | SQSTM1 | TUBULINE | RATIO |
| **3** | PDB SQSTM1+DOCK6- | 74948 | 339240 | 0.22092914 | 63468 | 339240 | 0.18708879 | 205988 | 343720 | 0.59929012 | 52704 | 1076582 | 0.04895493 | 927344 | 1063612 | 0.87188185 |
|  |  | 100368 | 374000 | 0.26836364 | 133598 | 374000 | 0.3572139 | 384207 | 239785 | 1.60229789 | 75392 | 890800 | 0.08463404 | 1292928 | 756704 | 1.70863112 |
|  |  | 55648 | 374616 | 0.14854678 | 85342 | 374616 | 0.22781195 | 239430 | 189696 | 1.26217738 | 241930 | 1129662 | 0.2141614 | 791198 | 718239 | 1.10158039 |
|  |  | 14800 | 22644 | 0.65359477 | 10192 | 22644 | 0.45009716 | 150008 | 65959 | 2.27426128 | 27477 | 1020935 | 0.02691356 | 350037 | 336616 | 1.03987036 |
|  |  | 13072 | 133731 | 0.09774847 | 6930 | 133731 | 0.05182045 | 112002 | 236061 | 0.47446211 | 22912 | 855779 | 0.02677327 | 996226 | 621300 | 1.60345405 |
|  |  | 35112 | 171665 | 0.20453791 | 5063 | 171665 | 0.02949349 | 107835 | 213836 | 0.50428833 | 13774 | 1021315 | 0.01348653 | 708183 | 582120 | 1.21655844 |
|  |  | 99826 | 374400 | 0.26662927 | 40503 | 374400 | 0.10818109 | 152961 | 601992 | 0.25409142 | 38610 | 1135017 | 0.03401711 | 949926 | 875490 | 1.0850221 |
|  |  | 34293 | 136312 | 0.25157726 | 12782 | 136312 | 0.09377017 | 55445 | 331170 | 0.16742157 | 22348 | 1057050 | 0.02114186 | 1066208 | 891384 | 1.19612647 |
|  |  |  |  |  |  |  |  |  |  |  |  |  |  |  |  |  |
|  | GEL2 | DOCK6 | VINCULIN | RATIO | DOCK5 | VINCULIN | RATIO | DOCK2 | VINCULIN | RATIO | RAC1 | TUBULINE | RATIO | SQSTM1 | TUBULINE | RATIO |
| 4 | PDB SQSTM1-DOCK6- | 290248 | 405460 | 0.71584867 | 46782 | 405460 | 0.11538006 | 309681 | 433782 | 0.71390929 | 263581 | 620620 | 0.42470594 | 1236570 | 1233700 | 1.00232634 |
|  |  | 298512 | 308730 | 0.96690312 | 4940 | 308730 | 0.01600104 | 140465 | 411048 | 0.34172408 | 55461 | 687120 | 0.08071516 | 1167950 | 1240381 | 0.94160585 |
|  |  | 23407 | 125305 | 0.18680021 | 8280 | 125305 | 0.06607877 | 267894 | 165127 | 1.62235128 | 21774 | 455552 | 0.04779696 | 461831 | 753756 | 0.61270623 |
|  |  | 118524 | 432038 | 0.27433698 | 18980 | 432038 | 0.04393132 | 145700 | 433222 | 0.33631718 | 74945 | 612028 | 0.12245355 | 659664 | 629244 | 1.04834373 |
|  |  | 12549 | 131130 | 0.09569892 | 37950 | 131130 | 0.28940746 | 13728 | 43344 | 0.31672204 | 7015 | 231232 | 0.0303375 | 576774 | 661941 | 0.87133748 |
|  |  |  |  |  |  |  |  |  |  |  |  |  |  |  |  |  |
|  |  |  |  |  |  |  |  |  |  |  |  |  |  |  |  |  |
|  | GEL 4 | DOCK6 | VINCULIN | RATIO | DOCK5 | VINCULIN | RATIO | DOCK2 | VINCULIN | RATIO | RAC1 | TUBULINE | RATIO | SQSTM1 | TUBULINE | RATIO |
| 5 | Healthy control | 81687 | 839880 | 0.09726032 | 108339 | 839880 | 0.12899343 | 329653 | 532060 | 0.61957862 | 87238 | 836072 | 0.10434269 | 927780 | 889878 | 1.04259236 |
|  |  | 124040 | 822198 | 0.1508639 | 97965 | 822198 | 0.11915013 | 533849 | 670286 | 0.79644958 | 21306 | 966875 | 0.02203594 | 606800 | 605377 | 1.0023506 |
|  |  | 145944 | 751764 | 0.19413539 | 97083 | 751764 | 0.12914026 | 400892 | 683316 | 0.5866861 | 33300 | 1136743 | 0.02929422 | 812764 | 737064 | 1.10270479 |
|  |  | 274344 | 866957 | 0.31644476 | 78715 | 866957 | 0.09079458 | 584237 | 821315 | 0.71134339 | 123900 | 984312 | 0.12587472 | 823691 | 828780 | 0.99385965 |
|  |  | 12218 | 65120 | 0.18762285 | 6643 | 65120 | 0.10201167 | 31416 | 210826 | 0.14901388 | 13420 | 339894 | 0.0394829 | 357043 | 437645 | 0.8158279 |
|  |  | 55767 | 40886 | 1.36396321 | 17475 | 40886 | 0.42740791 | 18352 | 78660 | 0.23330791 | 14076 | 146766 | 0.09590777 | 360360 | 453324 | 0.79492813 |
|  |  | 23016 | 17253 | 1.33402886 | 7216 | 17253 | 0.4182461 | 10296 | 85077 | 0.12101978 | 12376 | 834632 | 0.01482809 | 556465 | 506340 | 1.09899475 |
|  |  | 14784 | 323232 | 0.04573805 | 5000 | 323232 | 0.01546877 | 244110 | 574020 | 0.42526393 | 50025 | 1210077 | 0.04134034 | 1220947 | 863752 | 1.41353884 |

**Supplementary figure 1**: Full-lenthg Western-Blot gels of the SQSTM1, DOCKs and RAC1 protein expressions in mature osteoclasts. A: SQSTM1 expression. B: DOCK6 expression. C: RAC1 expression. D: DOCK5 expression. E: DOCK2 expression. Number regarding wells refers to participant categories in supplementary table 6.

A


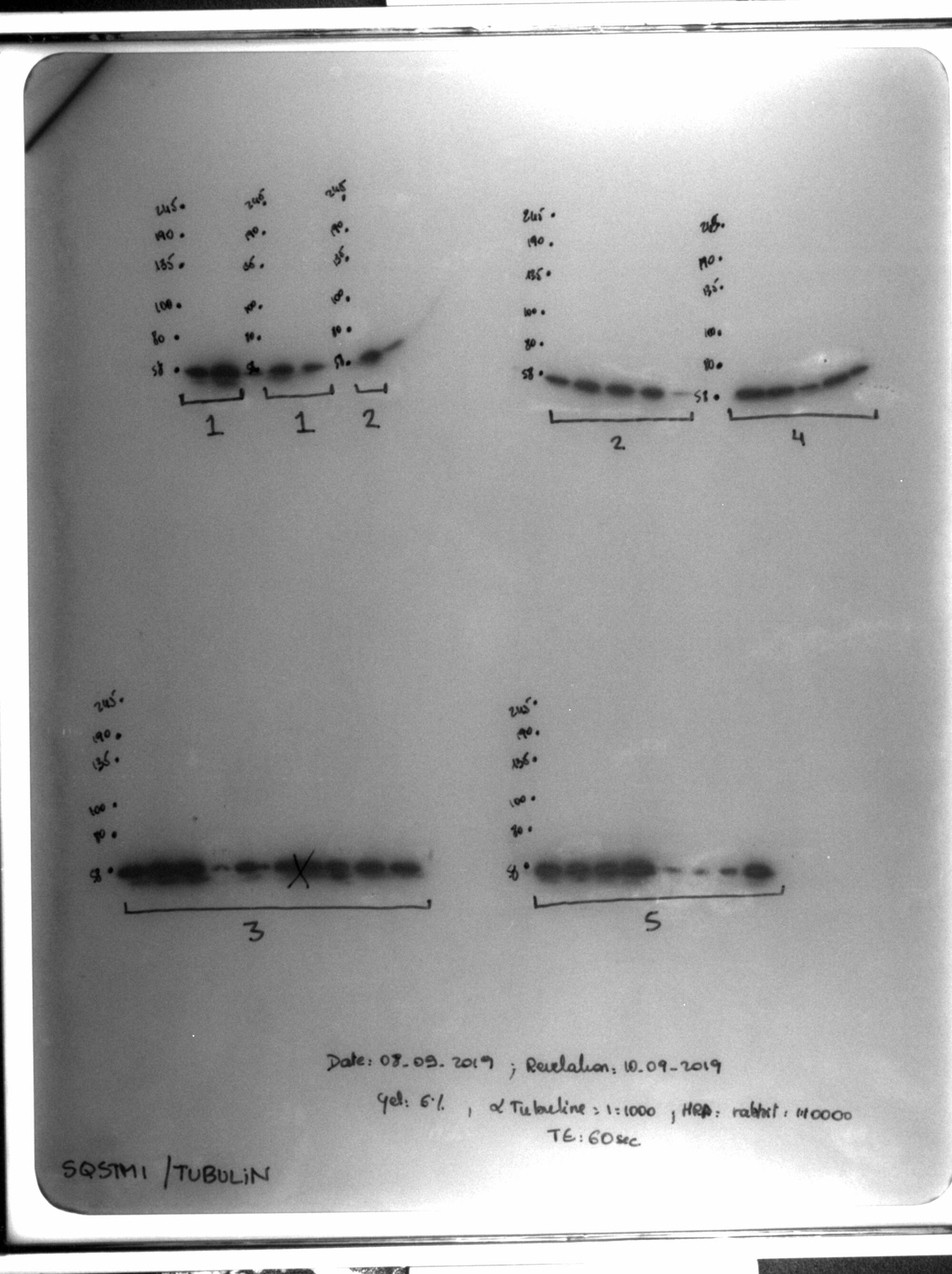

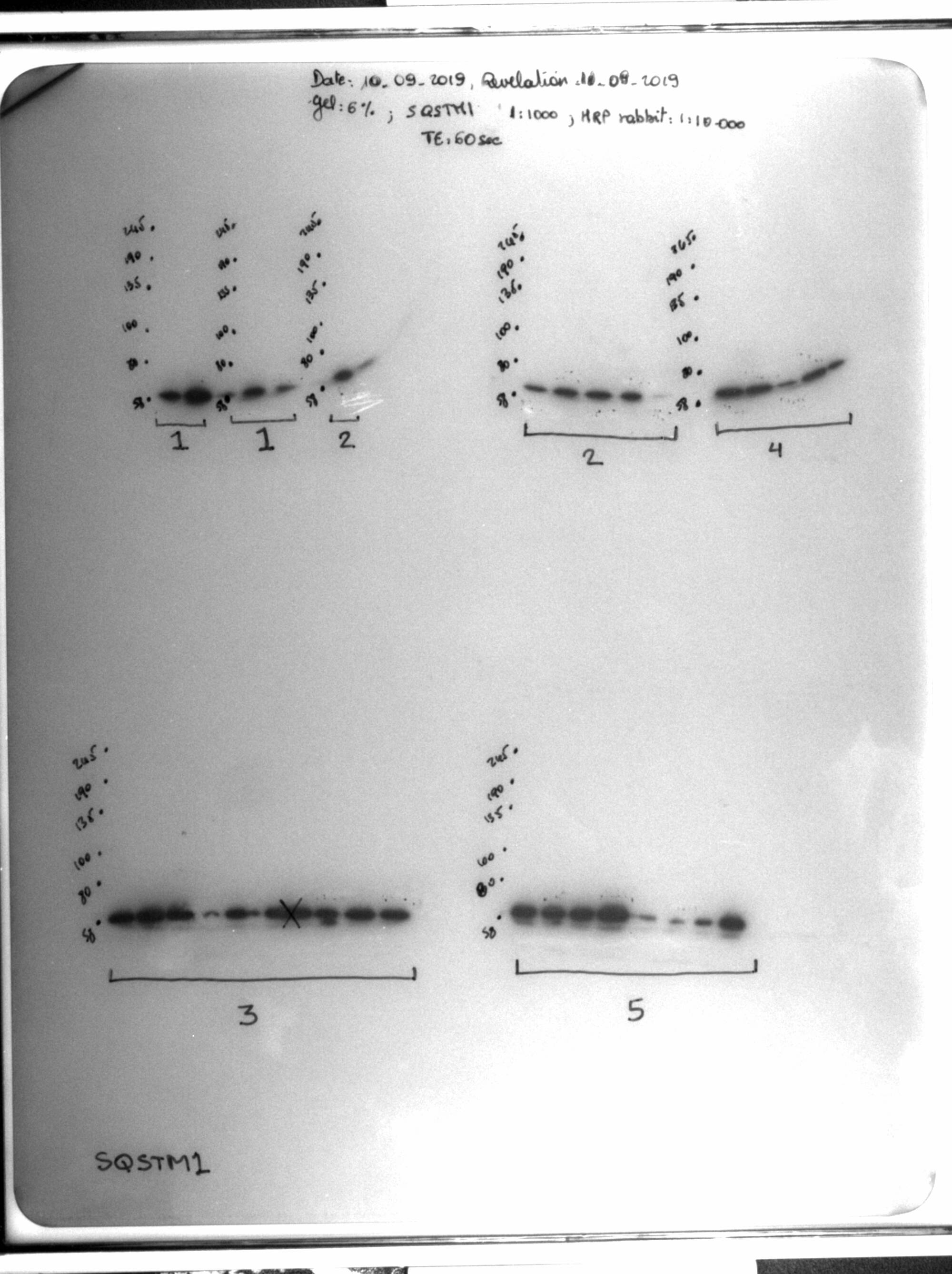


B


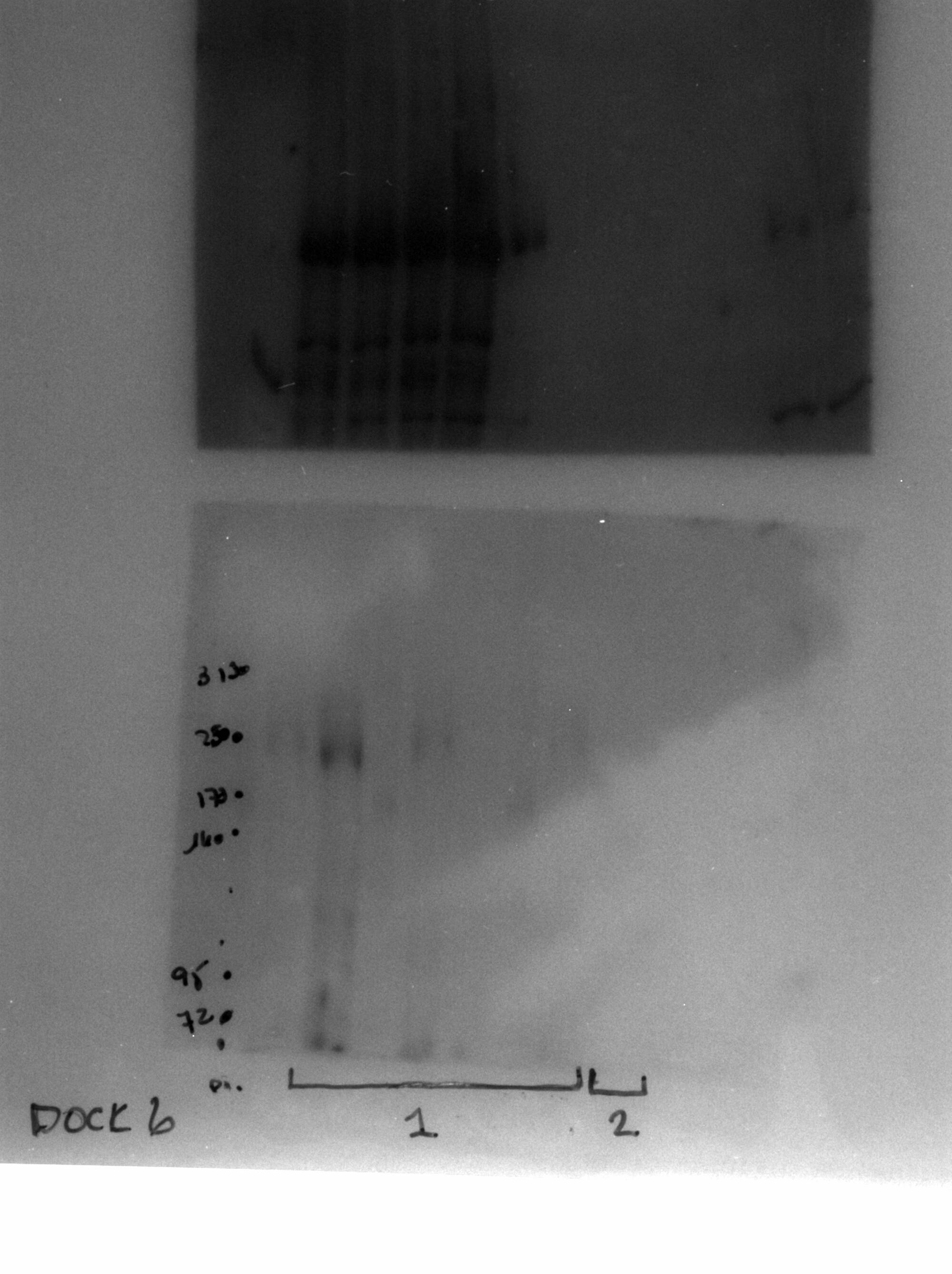

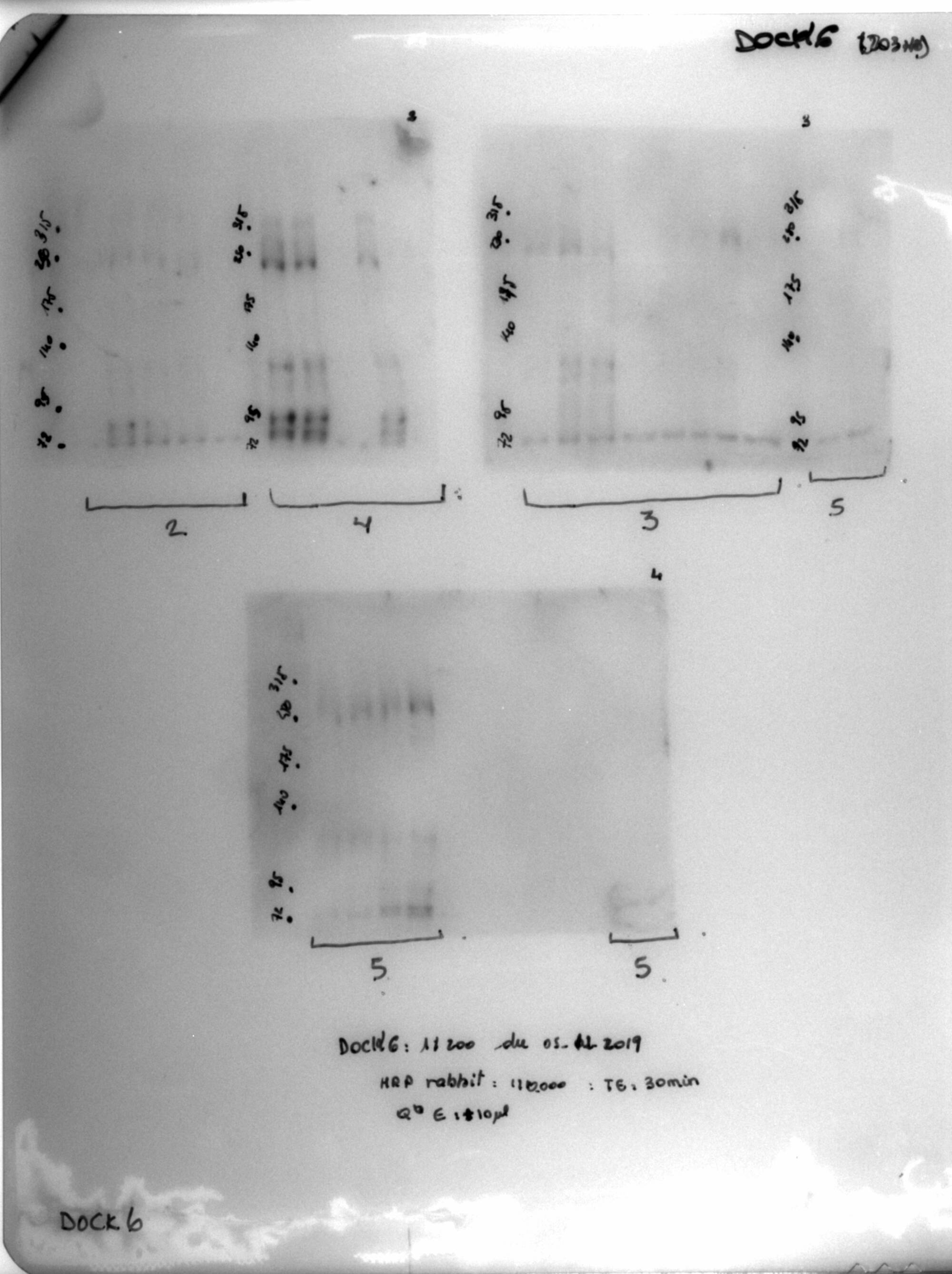


C


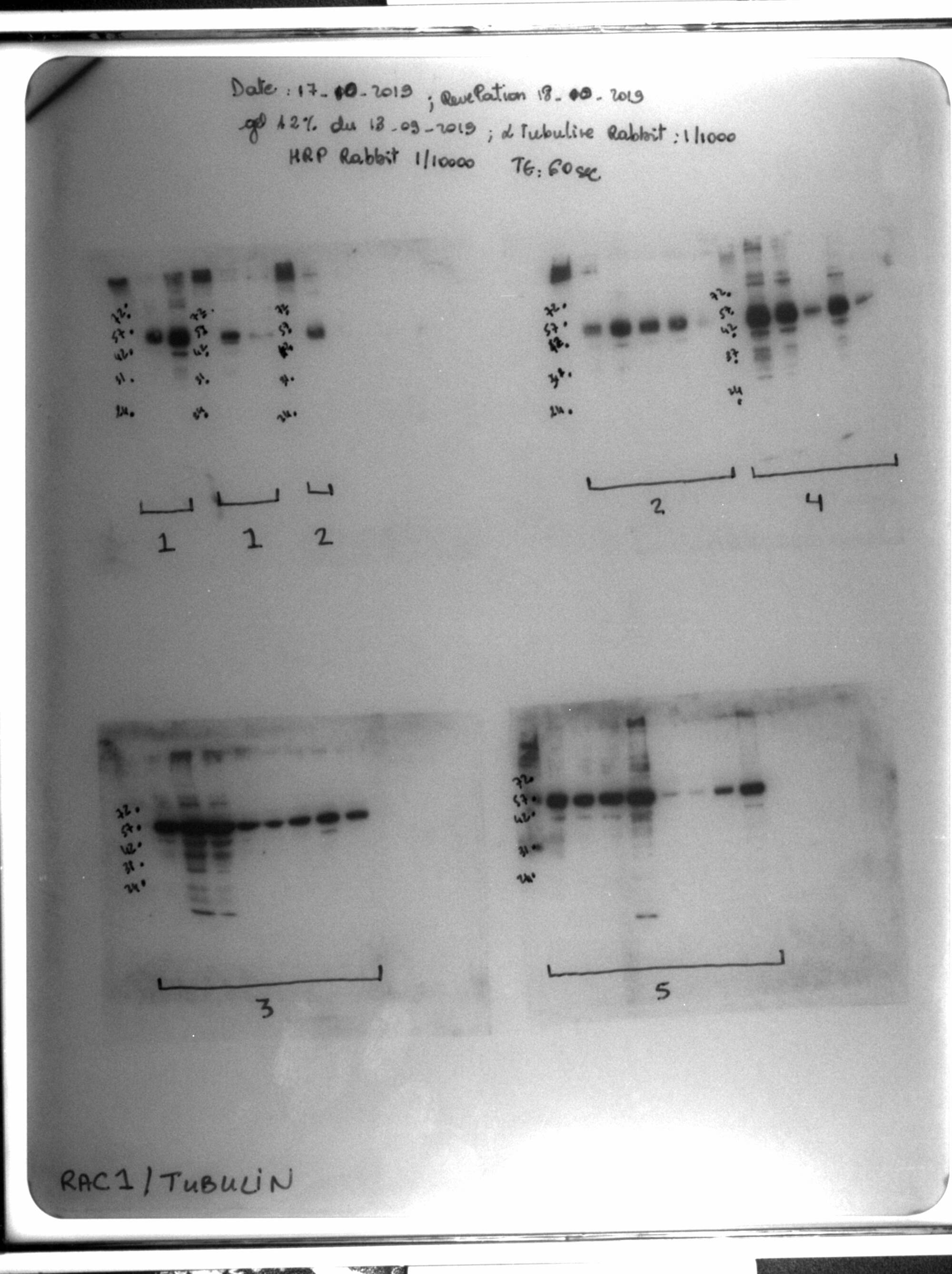

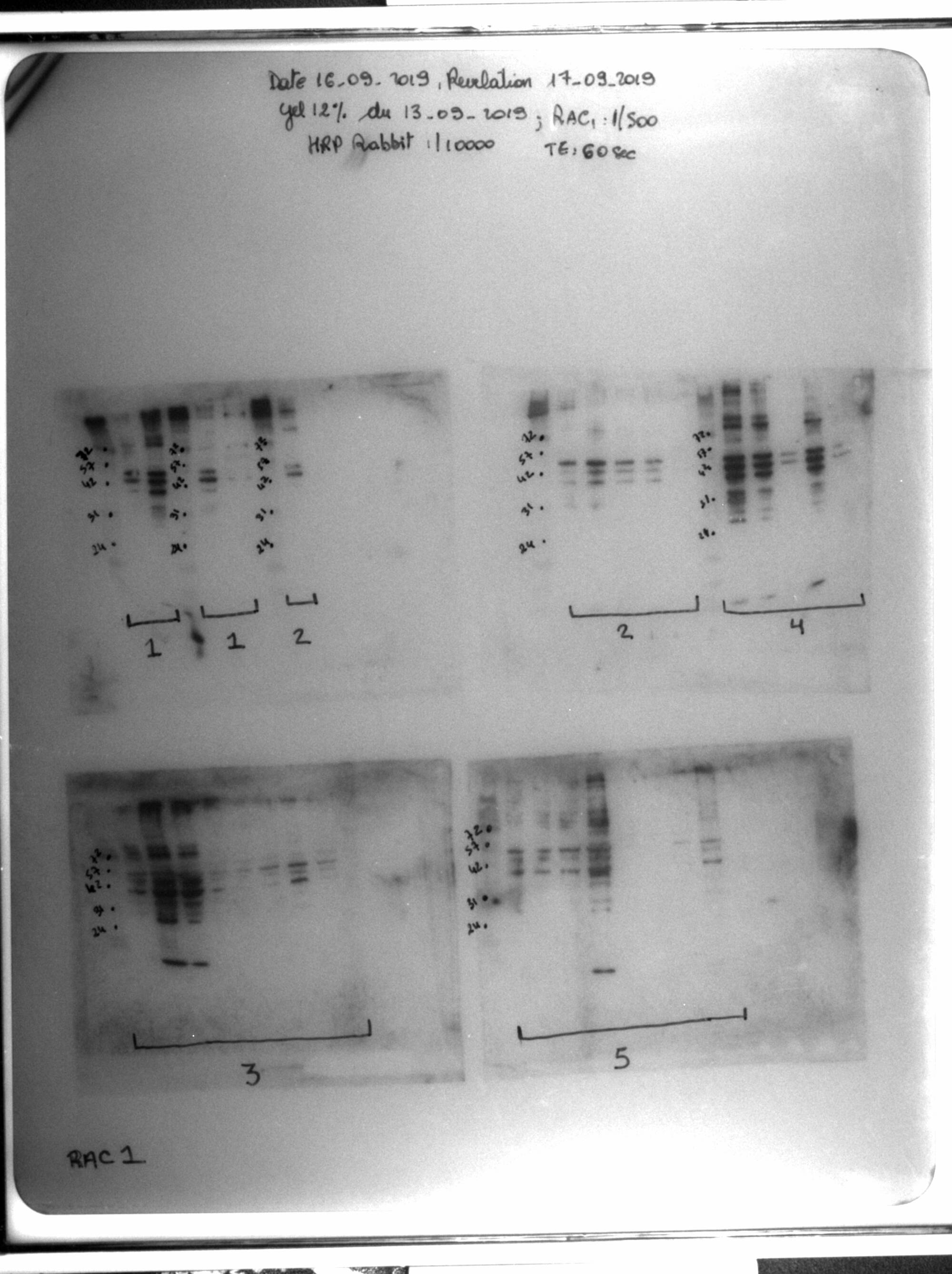


D


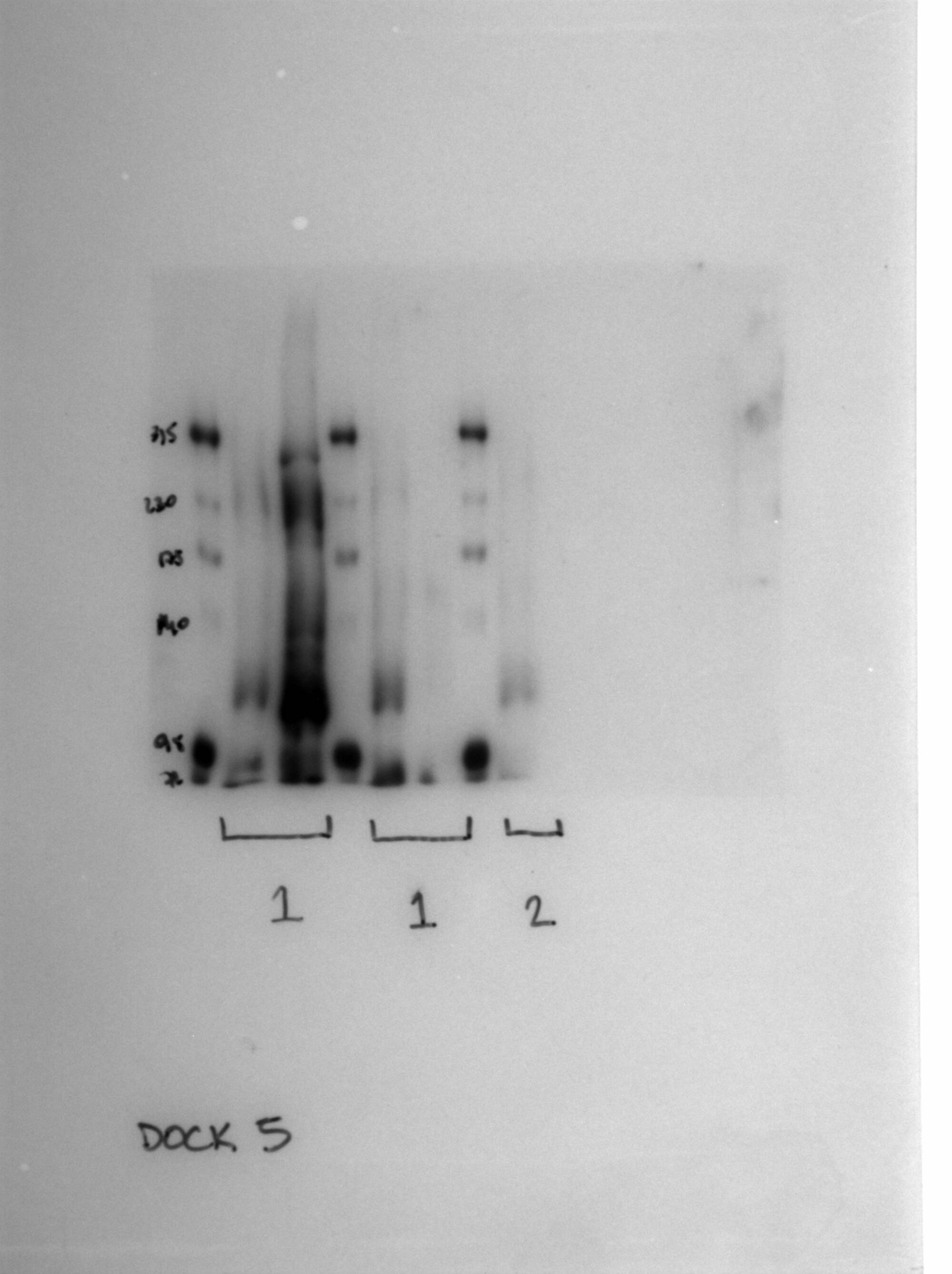

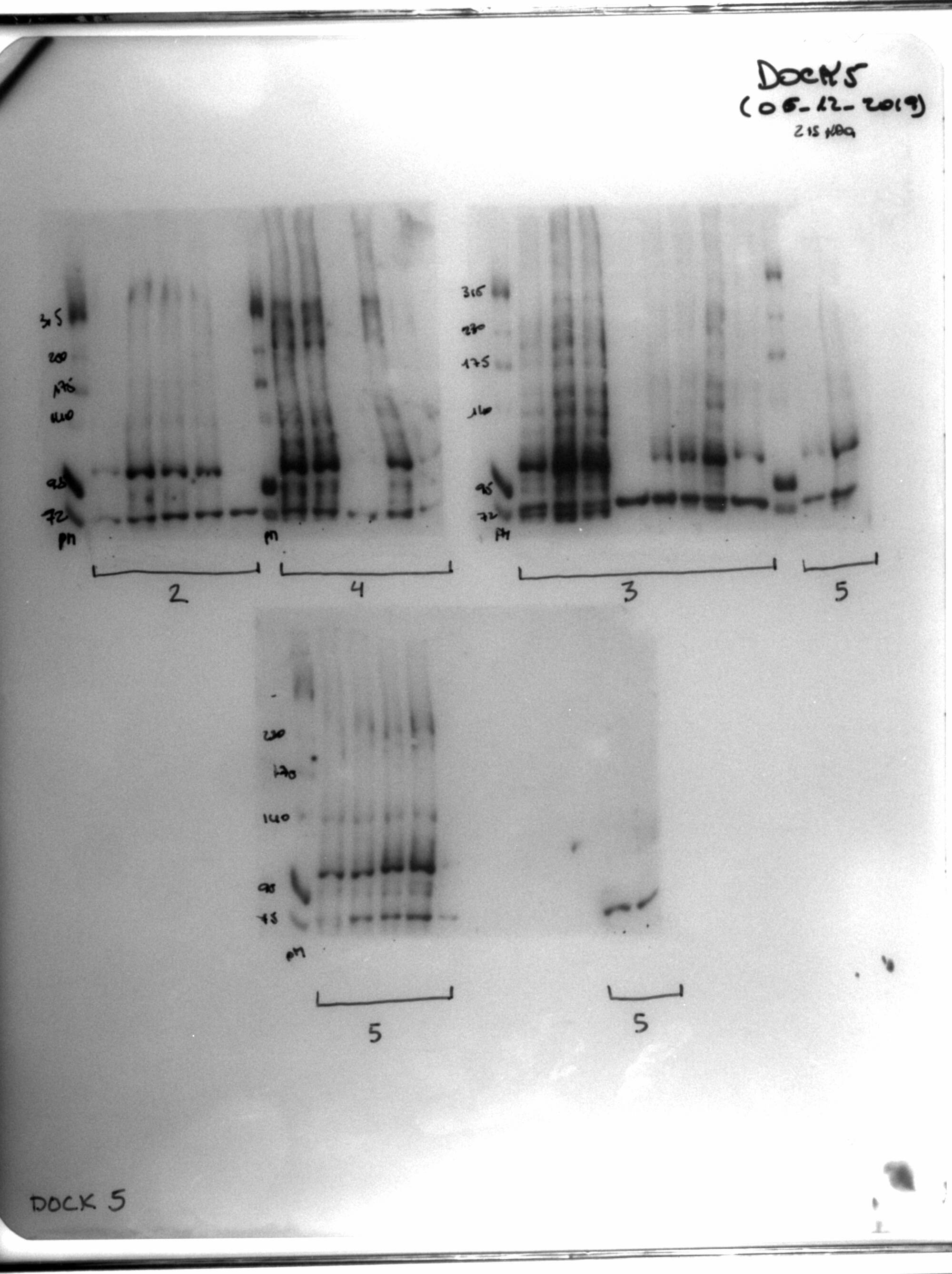

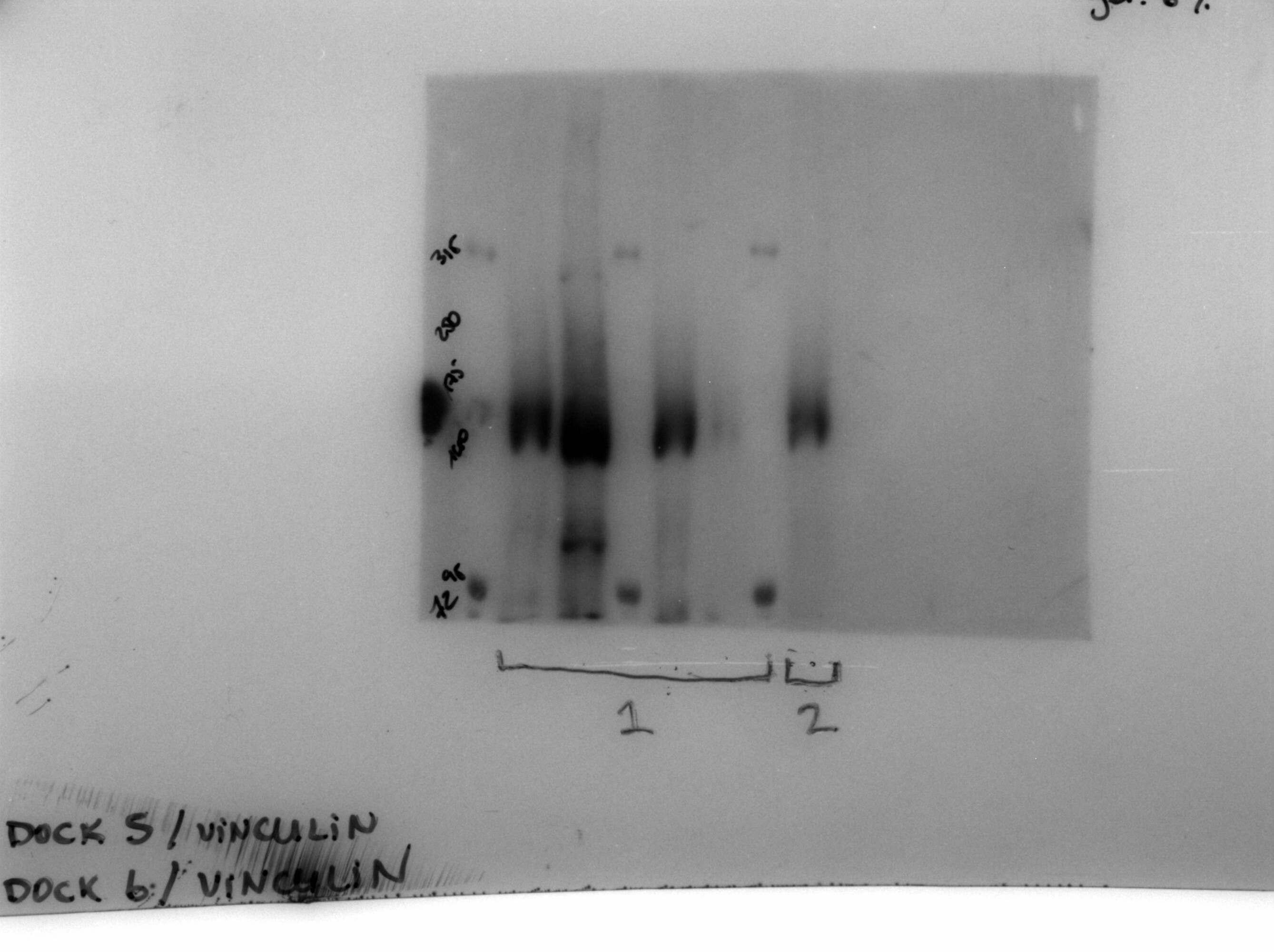

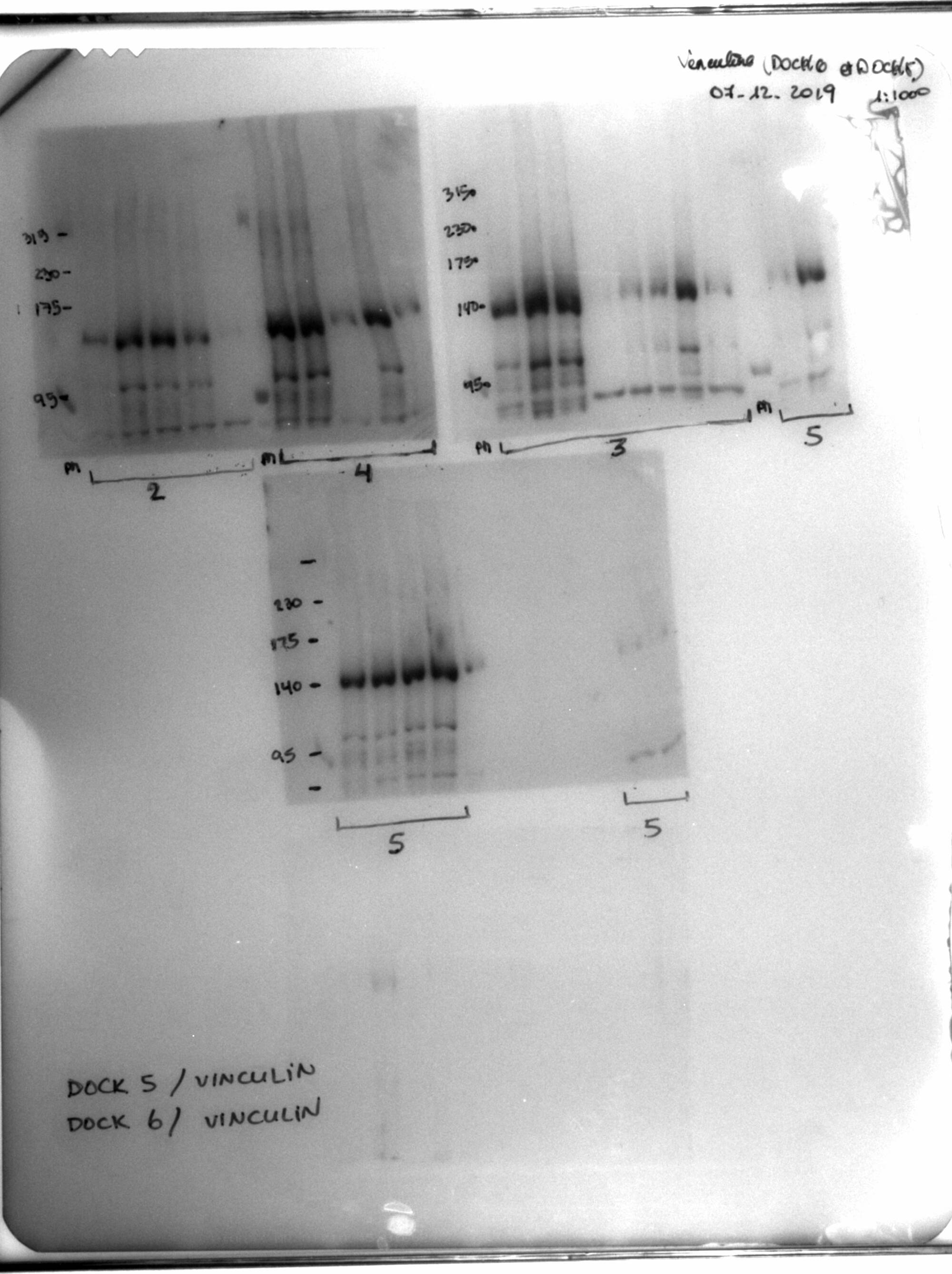


E


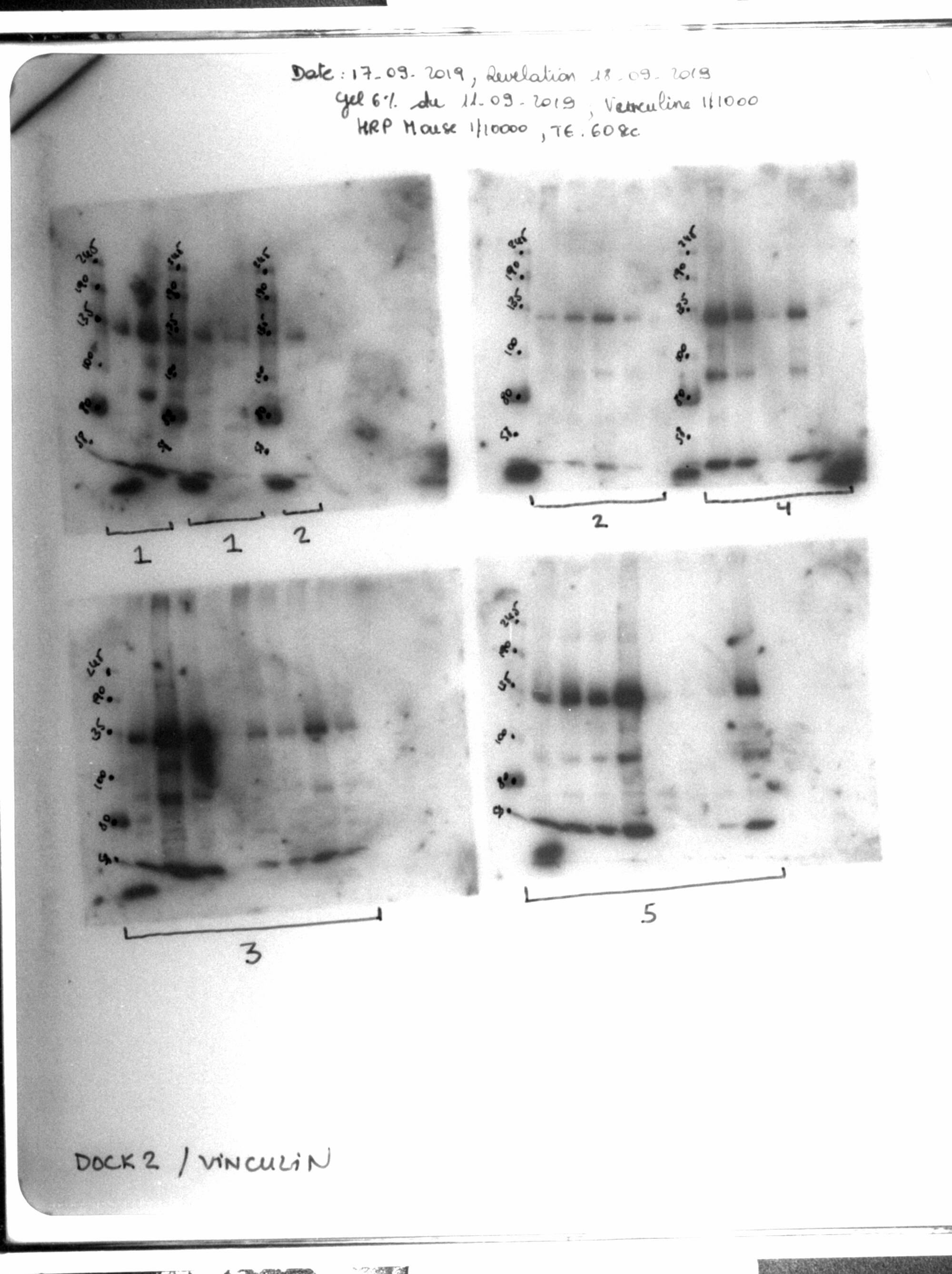

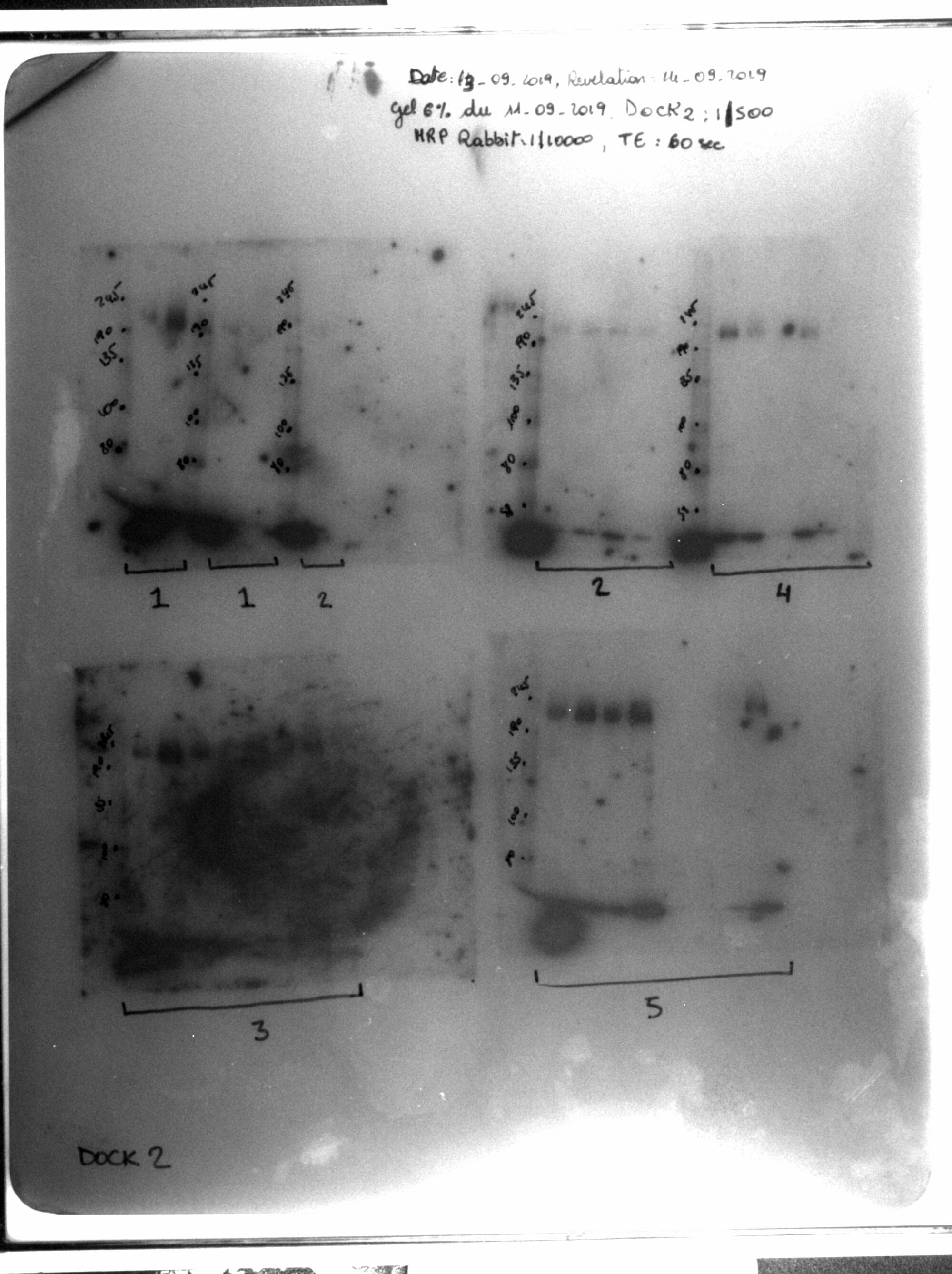

Supplement: Supplementary file 1 — Additional file 1. Supplementary data. [file 12920_2022_1198_MOESM1_ESM.doc]
